# Lipidomic Signatures Align with Inflammatory Patterns and Outcomes in Critical Illness

**DOI:** 10.21203/rs.3.rs-106579/v1

**Published:** 2021-01-08

**Authors:** Junru Wu, Anthony Cyr, Danielle S. Gruen, Tyler C. Lovelace, Panayiotis V. Benos, Tianmeng Chen, Francis X. Guyette, Mark H. Yazer, Brian J. Daley, Richard S. Miller, Brian G. Harbrecht, Jeffrey A. Claridge, Herb A. Phelan, Brian S. Zuckerbraun, Matthew D. Neal, Pär I. Johansson, Jakob Stensballe, Rami A. Namas, Yoram Vodovotz, Jason L. Sperry, Timothy R. Billiar, PAMPer study group

**Affiliations:** 1. Department of Surgery, University of Pittsburgh, Pittsburgh, Pennsylvania, USA.; 2. Pittsburgh Trauma Research Center, Division of Trauma and Acute Care Surgery, Pittsburgh, Pennsylvania, US.; 3. Department of Cardiology, The 3rd Xiangya Hospital, Central South University, Changsha, China.; 4. Eight-year program of medicine, Xiangya School of Medicine, Central South University, Changsha, China.; 5. Department of Computational and Systems Biology, University of Pittsburgh, Pittsburgh, Pennsylvania, USA.; 6. Joint CMU-Pitt PhD Program in Computational Biology, Pittsburgh, Pennsylvania, USA.; 7. Cellular and Molecular Pathology Program, University of Pittsburgh School of Medicine, Pittsburgh, PA, USA.; 8. Department of Emergency Medicine, Medicine, University of Pittsburgh, Pittsburgh, PA, USA.; 9. The Institute for Transfusion Medicine, Pittsburgh, Pennsylvania, USA.; 10. Department of Surgery, University of Tennessee Health Science Center, Knoxville, Tennessee, USA.; 11. Department of Surgery, Vanderbilt University Medical Center, Nashville, Tennessee, USA.; 12. Department of Surgery, University of Louisville, Louisville, Kentucky, USA.; 13. Metro Health Medical Center, Case Western Reserve University, Cleveland, Ohio, USA.; 14. Department of Surgery, University of Texas Southwestern, Dallas, Texas, USA.; 15. Section for Transfusion Medicine, Capital Region Blood Bank, Rigshospitalet, Copenhagen University Hospital, Copenhagen, Denmark.; 16. Department of Anesthesia and Trauma Center, Centre of Head and Orthopaedics, Rigshospitalet, Copenhagen University Hospital, Copenhagen, Denmark.; 17. Emergency Medical Services, The Capital Region of Denmark, Denmark.; 18. The PAMPer study group is detailed in Supplemental Acknowledgments.

## Abstract

Alterations in lipid metabolism have the potential to be markers as well as drivers of the pathobiology of acute critical illness. Here, we took advantage of the temporal precision offered by trauma as a common cause of critical illness to identify the dynamic patterns in the circulating lipidome in critically ill humans. The major findings include an early loss of all classes of circulating lipids followed by a delayed and selective lipogenesis in patients destined to remain critically ill. Early in the clinical course, Fresh Frozen Plasma administration led to improved survival in association with preserved lipid levels that related to favorable changes in coagulation and inflammation biomarkers. Late over-representation of phosphatidylethanolamines with critical illness led to the validation of a Lipid Reprogramming Score that was prognostic not only in trauma but also severe COVID-19 patients. Our lipidomic findings provide a new paradigm for the lipid response underlying critical illness.

## Introduction

Acute critical illness is a major healthcare burden and commonly leads to short and long-term morbidity and mortality^1,2^. Common causes of acute critical illness, including severe injury and infections, are among the leading causes of death worldwide^3^. Most recently, the COVID-19 pandemic has emerged as a major etiology for acute critical illness and death. Patients hospitalized for SARS CoV-2 infection that develop critical illness have mortality rates up to 39%^4^. For those that develop organ dysfunction, treatment options are limited and those targeting the host response are often nonspecific. Common features across these different etiologies of critical illness include dysregulated metabolism, an inflammatory “genomic storm”, immune suppression, and endothelial/ coagulation dysfunction^4–10^. The validation of accurate prognostic biomarkers and a better understanding of the pathobiology of acute critical illness would facilitate the identification of effective targeted therapies.

A limitation in the study of human critical illness is knowing the time of onset of the patient’s disease process^9^. This is especially true for infections for which time of onset is often unclear. In addition, serious infections are commonly seen on the background of other chronic diseases that can confound interpretation of results. Traumatic injury is one of the most common causes of acute critical illness and often occurs in otherwise healthy individuals. This, coupled to the fact that the time of onset of the acute disease process can be known with precision, makes trauma an attractive model for the study of the dynamic events leading up to acute critical illness.

Lipids comprise 30% of the body’s non-water mass and are not only a main component of cell membranes but also important energy substrates and signaling molecules^11^. Previous studies in critically ill humans provide evidence that lipolysis and lipogenesis are altered dramatically in acute critical illness. For example, circulating levels of glycerolipids, sphingolipids, phospholipids, and lyso-phospholipids vary from baseline in patients with acute critical illness^12–18^. However, a comprehensive assessment of the changes in circulating lipids that correlate with outcomes and markers of disease in acute critical illness is lacking.

To define the changes in the circulating lipidome associated with acute critical illness, we utilized a database and biobank established during the Prehospital Air Medical Plasma (PAMPer) Trial^19^. This prospective, multi-institutional randomized trial enrolled severely injured patients transported to level I Trauma Centers by helicopter. The trial demonstrated that administration of fresh frozen plasma (FFP) during transport improved 30-day survival when compared to standard-of-care, which does not include FFP in the pre-hospital setting. Because of this striking treatment effect, we hypothesized that early FFP administration would favorably impact circulating lipidomic patterns. Causal modeling was used to integrate the major changes in lipidomic profiles with immune mediator profiles and tissue injury/ coagulation markers observed after trauma and during critical illness. The lipidomic findings were further translated into a Lipid Reprogramming Score that was found to correlate highly with later patient outcomes. These findings were validated in a second trauma database and two publicly available databases that include critically ill COVID-19 patients, suggesting that some of the unique lipidomic patterns identified in this study may be generalizable to critical illness resulting from multiple etiologies.

## Results

### Lipid profiling of plasma from patients with severe trauma

To determine the dynamics changes in circulating lipids after severe injury in humans, we carried out a quantitative analysis of plasma lipid levels in samples obtained during the PAMPer trial^19^. This prospective, multi-institutional, pragmatic trial enrolled seriously injured humans suffering polytrauma at risk for hemorrhagic shock. Only patients that were transported by helicopter to a Level 1 trauma center were included and randomization took place in the pre-hospital setting. Patients in the treatment arm received two units of FFP initiated during helicopter transport, while the control group was assigned randomly to standard-of-care, which did not include FFP in the pre-hospital setting. The use of pre-hospital FFP was associated with a 9.8% reduction in 30-day mortality (p=0.03)^19^. A total of 193 of the original 523 patients were selected for lipidome analysis (Fig S1). This cohort included both non-survivors (n=72) and survivors (n=121) selected to represent the overall cohort. Samples were obtained at admission to the trauma center (0h) and at 24 and 72h after admission. Only the time 0h sample was obtained in the early non-survivors (n=51). A group of 17 non-fasting healthy subjects was used as controls for baseline values. The detailed demographic information of these patients is shown in Table 1. Since underlying medical conditions and medication history can influence circulating lipid profiles, we also provide this information (Table S4). Chronic health conditions and medications were rare in the trauma patient population and evenly distributed across the outcome groups (Table S1).

**Figure 1.**
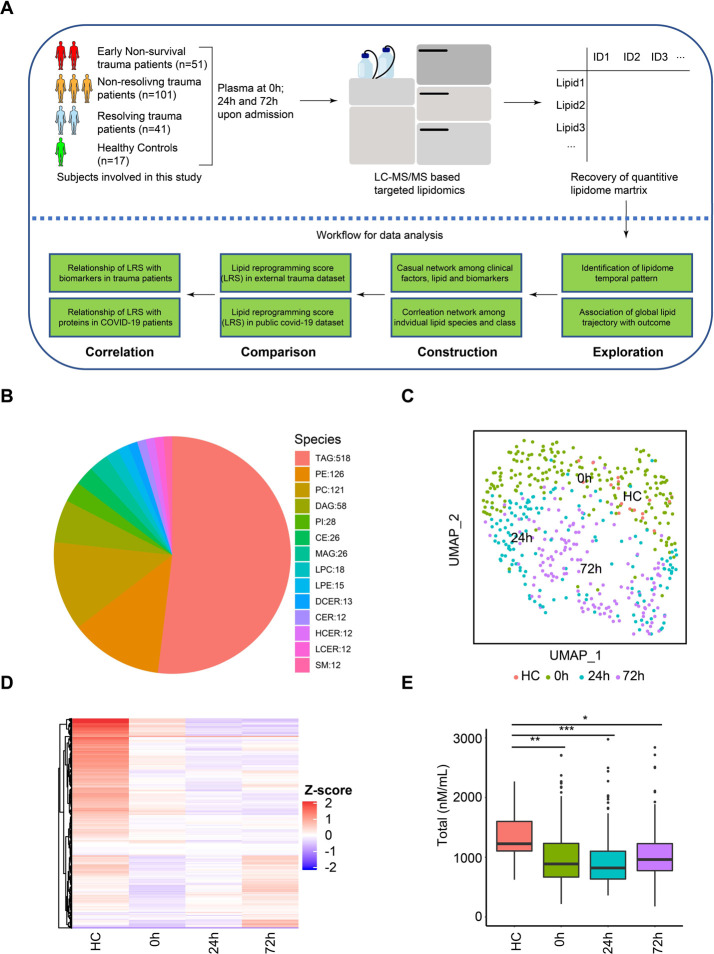
Temporal patterns in the circulating lipidome after severe trauma. **(A)** Scheme of overall analysis strategy. **(B)** Representation of 996 lipid species detected in the lipidomic platform grouped by classes. **(C)** Uniform Manifold Approximation and Projection (UMAP) plot shows the distribution of healthy subjects (n=17) and patients with trauma (n=193), grouped by sampling timepoints (0h, 24h, 72h after admission). **(D)** Heatmap shows relative levels of 996 lipid species for healthy subjects and trauma patients, grouped by sampling timepoints using z-score normalized concentrations. Lipid species are clustered by Hierarchical clustering. **(E)** Quantitative comparison of circulating total lipid concentration among healthy controls (HC) and trauma patients, grouped by sampling timepoints. Asterisks indicate statistical significance based on Kruskal-wallis test with post-hoc analysis of Dunn test. The p value was adjusted by the Benjamini-Hochberg method: *, < 0.05; **, < 0.01; ***, < 0.001. Box and whisker plots represent mean value, standard deviation, maximum and minimum values. Abbreviations: TAG, triacylglycerol; DAG, diacylglycerols; MAG, monoacylglycerols; PE, phosphatidylethanolamine; PC, phosphatidylcholine; PI, phosphatidylinositol; LPE, Lysophosphatidylethanolamine; LPC, Lysophosphatidylcholine; CER, Ceramides; HCER, hexosylceramides; LCER, lactosylceramide; DCER, dihydroceramides; CE, cholesterol ester.

The overall data analysis workflow is shown in Fig 1A. Liquid chromatography mass spectrometry (LC-MS) was used to carry out targeted lipidomic analysis on the plasma samples. In total, 996 lipids were quantified using internal standards. In the quality control analysis, the median relative standard deviation (RSD) for the lipid panel was 4%. Lipids are named according to sub-class and acyl chains detected. For example, PE (16:0/18:2) has a phosphatidylethanolamine (PE) backbone and two acyl chains comprised of palmitic acid (C16:0) and linoleic acid (C18:2). The representation of lipids from 14 sub-classes is shown in Fig 1B. Triglyceride (TAG) (glycerol backbone + three acyl chains) was the most abundant lipid class identified in the plasma (n=518). Phosphatidylethanolamine (PE), phosphatidylcholine (PC), and diacylglycerols (DAG) all containing 2 acyl chains were the next most abundant classes (n=128, 121, 58 respectively).

We first explored the dynamic changes in the global pattern of the circulating lipidome in trauma patients. Uniform Manifold Approximation and Projection (UMAP) is a non-linear method for dimension reduction that can identify the global structure of multi-dimensional data. In Fig 1C, each dot represents a single subject and the distance between dots in the UMAP plot reflects the global similarity/ differences in overall lipid profiles between samples^20^. We observed that trauma patients at 0h were quite dispersed and partially overlapping with healthy subjects, suggesting an early and rapidly evolving response pattern immediately post-injury. There was excellent separation across the three time points on UMAP, underscoring the role of time in the major changes in lipid patterns after trauma.

To depict the differences between the healthy controls and patients across time, we projected relative levels of all lipids assayed on a heatmap (Fig 1D). Compared to healthy controls, most lipid species were persistently lower after trauma. This dramatic shift between healthy controls and injured humans was also observed when total lipid concentrations were compared (Fig 1E).

### Association between lipidome pattern and outcome of trauma patients

**Figure 2.**
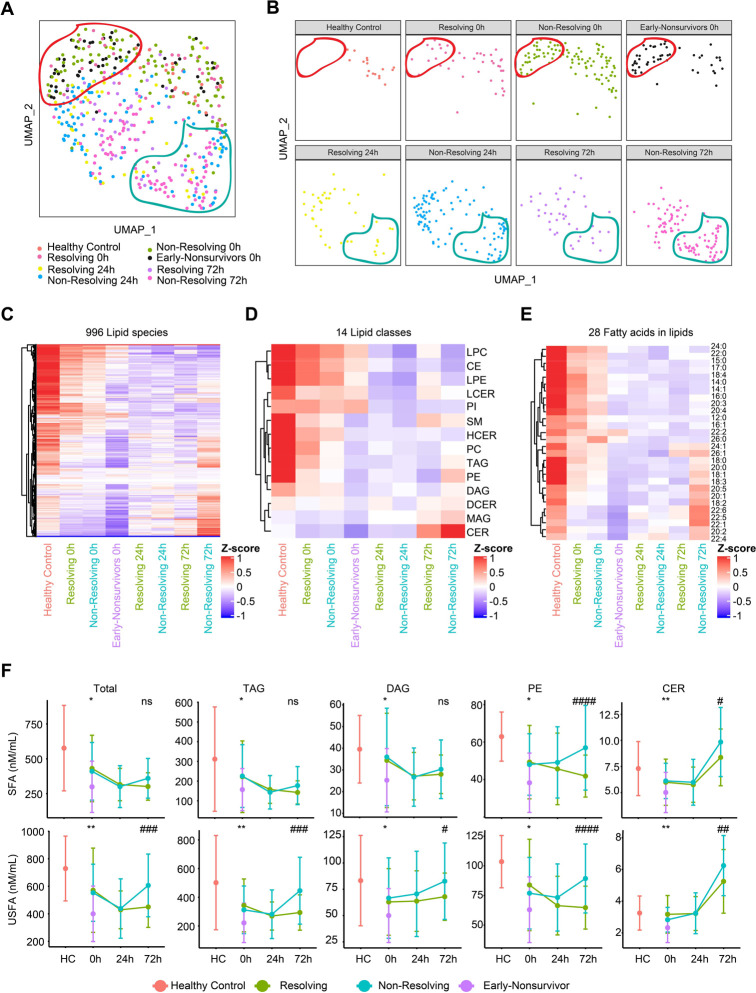
Association between temporal patterns of the circulating lipidome and outcome **(A-B)** Uniform Manifold Approximation and Projection (UMAP) plot shows the distribution of healthy control subjects (n=17) and trauma patients (n=193), grouped together **(A)** and separated **(B)** by outcome and sampling timepoints. **(C-E)** Heatmaps show relative levels of 996 lipid species **(C)**; 14 lipid classes **(D)** and 28 fatty acids labeled by carbon number: double bonds **(E)** for healthy subjects and trauma patients, grouped by outcome and sampling timepoints. z-score represents normalized concentrations. Rows are clustered by method of hierarchical clustering. **(F)** Quantitative comparison of circulating total lipid concentrations among healthy controls (HC) and trauma patients. Lipids are grouped by classes and fatty acids (saturated or unsaturated) identified as the acyl chains in the lipid classes. Patients are grouped by outcome and sampling timepoints. Center dots and error bars represent median value and median absolute deviation, respectively. SFA: saturated fatty acid; USFA: unsaturated fatty acid. Asterisks indicate statistical significance based on Kruskal-wallis test among 3 groups at 0h with post-hoc analysis of Dunn test. The P value was adjusted by Benjamini-Hochberg method: *, < 0.05; **, < 0.01. Number sign indicates statistical significance based on 2-way AVOVA test of time-series analysis of resolving and non-resolving groups. Pairwise Comparisons were conducted by Estimated Marginal Means test. The P value was adjusted by Benjamini-Hochberg method: #, < 0.05; ##, < 0.01; ###, < 0.001, #### < 0.0001.

We next investigated the association between the circulating lipidome and patient outcomes. The three outcomes used for this analysis included (1) early non-survivors (death within 3 days of admission), (2) non-resolving patients (survivors with duration of intensive care unit [ICU] stay ≥7 days or patients that died after day 3 following admission), and (3) resolving patients (survivors with duration of ICU stay <7 days). UMAP plots of the global lipidomic patterns indicated enrichment of early non-survivors in the region encircled in red at 0h and an enrichment of the non-resolving patients in the region encircled by the blue line at 72h after admission (Fig 2A&B). Furthermore, we observed a dramatic drop in the levels of nearly all major lipid species at 0h for early non-survivors compared to the other patient groups or healthy controls (Fig 2C). Patients in both the resolving and non-resolving groups at 0h also exhibited a drop in most lipid species compared to healthy controls, but not to the degree seen in the non-survivors. Patients in the resolving group exhibited a persistent suppression in most lipids at 24 and 72h (Fig 2D&E). Remarkably, patients in the non-resolving group at 72h demonstrated an increase in a subset of lipids. Further characterization of lipid class and fatty acid types indicated that all 14 classes, including both saturated and unsaturated fatty acids, were suppressed at 0h. However, there was selective elevation of TAG, DAG, PE, and ceramides (CER) at 72h in the non-resolving cohort. A quantitative time-series analysis showed that total lipid levels were higher at 72h in the non-resolving patients and that unsaturated fatty acids predominated in TAG and DAG, while PE and CER contained a mixture of saturated and unsaturated fatty acids (Fig 2F). Our findings point to a rapidly evolving pattern in the circulating lipidome after severe injury that includes a loss of all classes of lipids in the circulation after injury. This process is exaggerated in patients that die early. Furthermore, there is a selective increase in four lipid classes by 72h in patients that remain critically ill or die later in their clinical course.

**Figure 3.**
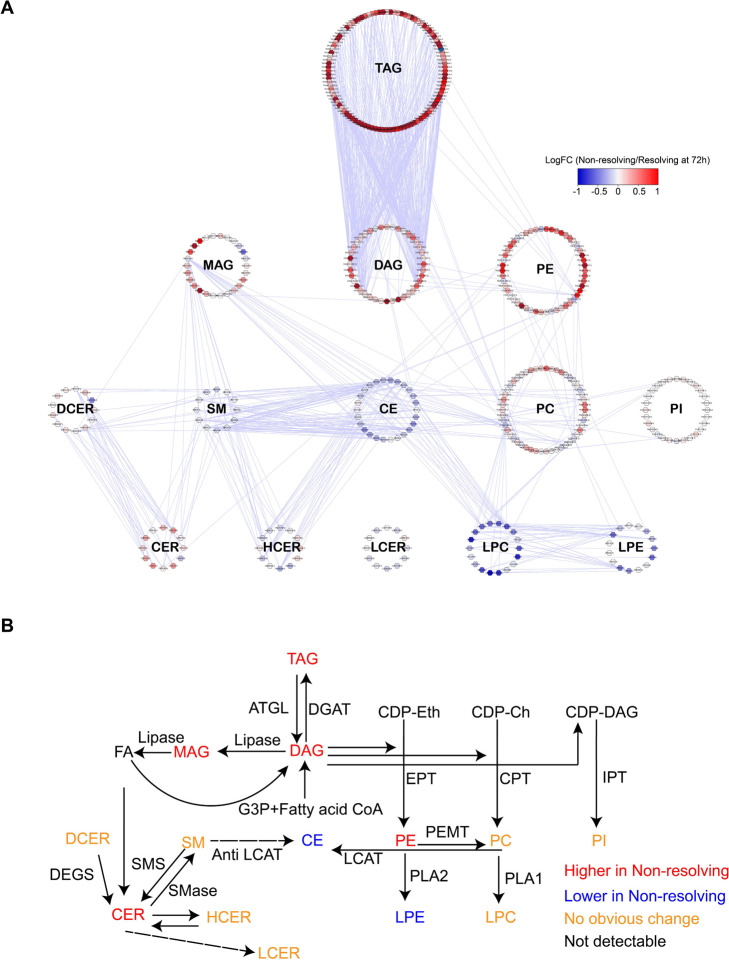
Lipidome network in non-resolving trauma patients at 72h **(A)** Correlation network among 412 lipids from 14 classes represented in the lipidomic dataset. Each dot indicates a lipid and is depicted in a circle if it belongs to one class. Highly correlated (Pearson coefficient > 0.7) lipids are represented by edges. Only inter-class correlations are shown. Relative levels are color coded for each lipid species between non-resolving and resolving trauma patients at 72h after admission. **(B)** Synthesis pathways for the 14 lipid classes summarized from published literature. Colored by differential levels of each lipid class between non-resolving and resolving trauma patients at 72h admission. Abbreviations: ATGL, Adipose Triglyceride Lipase; DAGT, diacylglycerol acyltransferase; G3P, glycerol-3-phosphate; CDP-Eth, Cytidine diphosphate-Ethanolamine; CDP-Ch, Cytidine diphosphate-Choline, CDP-DAG, Cytidine diphosphate-diacylglycerol, EPT, Ethanolamine phosphotransferase; CPT, Choline phosphotransferase; IPT, inositol phosphatidyltransferase. PLA, phospholipase A; PEMT, Phosphatidylethanolamine N-methyltransferase; LCAT, cholesterol acyltransferase; SMS, Sphingomyelin Synthase; SMase, Sphingomyelin phosphodiesterase; DEGS, dihydroceramide desaturase.

To better visualize the changes in individual lipid species, we created a correlation network of 412 lipids shown to differ between the resolving and non-resolving patients at 72h (Fig 3A). Only highly correlated relationships between each connected lipid pair in the correlation network (Pearson correlation coefficient r >0.7) were kept. Lipids within each class were well correlated with each other. Furthermore, we identified a unique relationship for the inter-class networks. The dominant type of lipids that increased from baseline in non-resolving patients were from the DAG-TAG and PE classes (Fig 3A). DAG and PE are produced in the liver and kidney by the conversion of the same precursors (fatty acid-CoA and L-glycerol-3-phosphate), first to phosphatidic acid and then either DAG or PE. PE and other glycerophospholipids are generated by the addition of headgroups (e.g. ethanolamine for PE or choline for PC) while TAG is synthesized from DAG by the addition of a third acyl group by acyl transferase. Also evident from the figure is the suppression of the cholesterol (CE) and LPE families of lipids. The interconnections between biochemical pathways involved in the synthesis of the lipid classes are shown in Fig 3B. The pathways are color coded to show how these pathways relate to the changes in lipid levels in the non-resolving group.

We next examined the impact of injury severity reflected by injury severity scores (ISS) on lipid levels and profiles. Patients were separated into minimal (ISS<10), moderate (ISS 10–25), or severe (ISS ≥ 25) injury (Fig S2A). Exploration of the lipid profiles by either UMAP or heatmap demonstrated no major impact of ISS on the post-injury lipid patterns (Fig S2B). We also observed poor correlation between ISS and total lipids concentrations of either saturated or unsaturated fatty acids (Fig S2C&D, 0h timepoint shown). Thus, while injury induces major changes in the circulating lipidome, in this cohort of patients with shock on presentation, ISS alone does not associate with lipid patterns.

### Pre-hospital FFP enhances lipid levels early after severe injury

**Figure 4.**
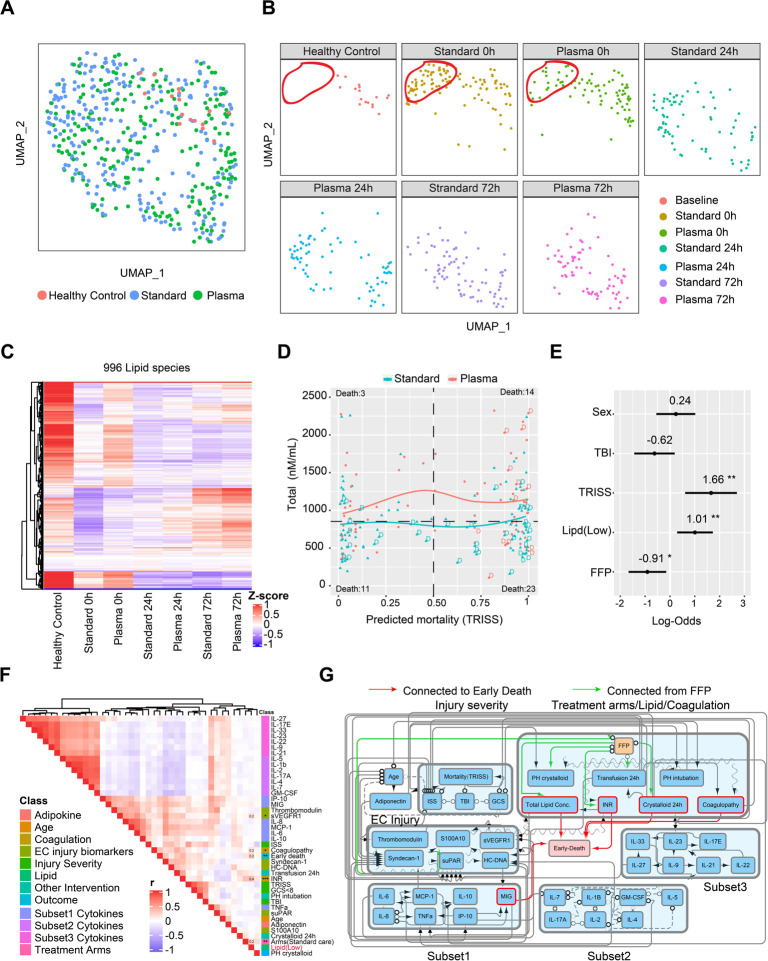
Potential casual effect for fresh frozen plasma (FFP), Lipid concentration and early mortality **(A-B)** Uniform Manifold Approximation and Projection (UMAP) plot shows the distribution of healthy subjects (n=17) and patients with trauma (n=193) (A), separated by treatment arms with sampling timepoints (B). **(C)** Heatmap show relative levels of 996 lipid species for healthy subjects and trauma patients, grouping by treatment arms and sampling timepoints. Exp, z-score normalized concentration. Rows are clustered by hierarchical clustering. **(D)** Relationship of predicted mortality and total lipid concentration at 0h upon admission. Trauma patients are grouped by treatment arms; tendency lines are modeled by loess methods for 2 groups separately, dash line in the x-axis means 0.5 and y-axis means the median concentration. D indicates patients who died less than 72h after admission. **(E)** Forest plot showing log odds ratios from logistical regression of clinical factors; Lipid concentration; FFP effect for early-nonsurvivors versus others. **(F)** Correlation heatmap showing correlation among cytokines, biomarkers, clinical variables, total lipid concentration and outcome. r: Spearman correlation coefficient. **(G)** Casual network among factors in (E) constructed by FCI (see also methods). The presence of “edges” or connections between nodes in the graph correspond to conditional dependencies relationships. Orientations in the causal network indicate what can be inferred about the cause-effect relationships between variables in the dataset. A directed edge A --> B indicates that A is a cause of B (i.e., a change in A is expected to affect a change in B). A bidirected edge A <-> B indicates that there is unmeasured confounder affecting both A and B. A partially directed edge A o-> B indicates that B is not a cause of A, but it is unclear whether A is a cause of B or if there is a latent confounder that causes both A and B. An undirected edge A o-o B indicates that we cannot make inferences about the causal orientation of that edge. Abbreviations: TRISS, Trauma and injury severity score; FFP, Fresh frozen plasma; TBI, traumatic brain injury; ISS, injury severity score; GCS, Glasgow coma score; PH; Prehospital; INR, international normalized ratio. Asterisks in (E) indicate statistical significance in multi-variable logistic regression model: *, < 0.05; **, < 0.01. Asterisks in (F) indicate statistical significance for correlation coefficient. P-values are approximated by using the t distributions: *, < 0.05; **, < 0.01; ***, <0.001.

The key observation of the PAMPer trial was the demonstration that initiating FFP administration in the pre-hospital setting reduced early mortality when compared to standard care^19^. To assess for an impact of FFP, we compared lipid profiles in patients in the treatment arm to those in the standard-of-care arm. UMAP plots demonstrated a skewing in the lipid profiles towards the healthy controls in the FFP treatment group at 0h (Fig 4A&B). However, the impact of pre-hospital FFP on lipid profiles was seen to dissipate at 24 and 72h, with no difference in lipid levels or patterns between the FFP and standard-of-care groups at these time points. Both the qualitative and quantitative analysis revealed that patients receiving FFP had less of a drop in the levels of most classes of circulating lipids at time 0h, with a selective preservation of TAG, DAG, and MAG (Fig 4C, Fig S4A). We then assessed the relationship between the predicted mortality, calculated from the Trauma and Injury Severity Score (TRISS), and lipid levels in the two cohorts (Fig. 4D). Average lipid levels were higher in the FFP group across all TRISS values. All unexpected deaths (low TRISS Score: predicted mortality rate less than 50%) were in the standard-of-care patients and 11/14 had lipid levels below the mean for the overall cohort. Deaths seen in the FFP group were limited to those with a high expectation for death for all except one patient (high TRISS Score: predicted mortality rate of greater than 75%). A Forest plot of log-odds ratios from multi-variable logistical regression is shown in Fig. 4E. This analysis revealed that lower lipid levels at 0h significantly favored mortality within the first 72h while FFP administration favored survival. Only TRISS had a higher association with early mortality than FFP or lipid levels even when traumatic brain injury (TBI) and sex were added to the model.

We next carried out correlation analysis to identify the factors that associate with circulating lipid levels in the early response to severe injury. Included in the analysis were 21 inflammatory and immune mediators, 6 markers of endotheliopathy/ tissue injury, and 2 measures of coagulation abnormalities, all measured at time 0h. Also included in the analysis were typical measures of injury severity and interventions associated with adverse outcomes. Interestingly, the mediators segregated into three subsets, each with strong internal correlation (Fig 4F). These included a subset represented by pro-inflammatory cytokines and chemokines that mostly positively correlated with early death, injury severity, endotheliopathy, and abnormal coagulation (Subset 1: IL-6, IL-8, IL-10, MCP-1/CCL2, IP-10/CXCL10, and MIG/CXCL9) and two subsets that correlated inversely with the pro-inflammatory mediators and adverse outcomes including, mediators associated with type 2 and 3 immune responses (Subset 2: IL-2, IL-4, IL-5, IL-7, IL-17A, and GM-CSF) and mediators associated with either tissue protection/ repair or lymphocyte regulation (Subset 3: IL-9, IL-22, IL-25, IL-27, IL-33 and IL-21, IL-23). The relationships between these three mediator subsets remained mostly consistent at 24 and 72h (sFig. 7A&B). However, low lipid levels at time 0h positively correlated only with standard-of-care, early death, coagulation abnormalities and the endotheliopathy marker, sVEGFR, and not with any of the mediator subsets (Fig 4F).

We next used probabilistic graphical models for mixed data types^21,22^ to infer potential direct (cause-effect) relationships within the multi-modal observational data included in Figure 4F. These features were loaded into the algorithm and nodes and edges projected onto a graph with early mortality as the endpoint of interest (Fig. 4G). The α-value of 0.2 for the conditional independence tests of the algorithm was selected using nested leave-one-out cross-validation to select the model with the best predictive performance of patient outcome (see Methods). Circulating lipid concentrations, coagulopathy (including INR), volume of crystalloid used in first 24h and the pro-inflammatory mediators (via MIG) were identified as direct casual factors contributing to early death (demonstrated by red arrows). The sequential edges connected FFP administration to circulating lipid concentrations, coagulopathy, INR, and volume of crystalloid used in first 24h. These connections indicated a potential mixed causal relationship linking FFP with all these factors and fewer early deaths. Other features known to be important to early mortality, including patient and injury characteristics, endothelial and tissue injury, and subset 2 and 3 mediators were indirectly linked to outcomes. Thus, correlation analysis and causal modeling related an interaction between INR and lipid concentration to early death and identified a direct impact of FFP on both of these causative factors.

### Validation of outcome-based changes in the plasma lipidome in trauma and patients with critical illness due to COVID-19

**Figure 5.**
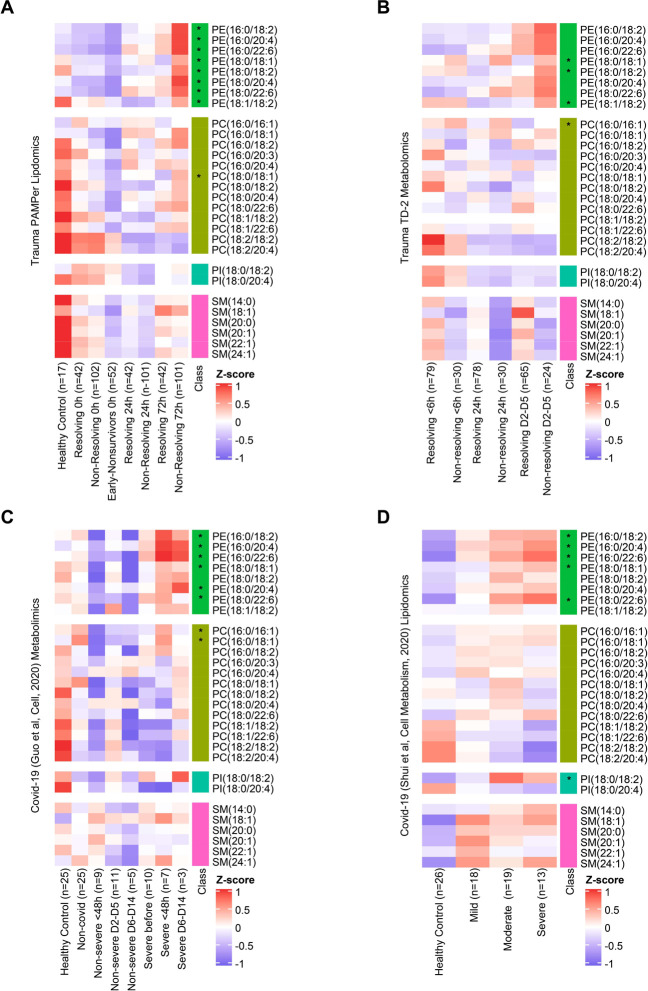
Comparison of temporal patterns of common lipids for patients with trauma or COVID-19 **(A-D)** Heatmaps show the relative levels of 29 common lipid species from four major classes across patients. Data comes from trauma patients from PAMPer lipidomics dataset **(A)** and TD-2 untargeted metabolomics dataset **(B);** COVID-19 patients from untargeted metabolomics dataset (Guo et al Cell, 2020) **(C)** and lipidomics dataset (Shui et al, Cell metabolism, 2020) **(D)**. Patients are grouped by outcome and sampling timepoint (except for D). Asterisks indicate lipids with statistical significance (p value <0.05) and log2 fold change >0.4 by Wilcoxon Rank Sum test between non-resolving and resolving trauma patients at 72h (A); non-resolving and resolving trauma patients at D2–D5 (B); severe and non-severe Covid-19 patients (C); severe and mild Covid-19 patients (D).

To further generalize our findings of outcome-associated changes in circulating lipids to other trauma datasets and causes of acute critical illness, we analyzed a separate trauma dataset^23^ (Trauma dataset-2:TD-2, n=86) and two public datasets derived from COVID-19 patients^16,17^. To assist with the comparison between these trauma and COVID-19 datasets, we set the 0 timepoint in the COVID-19 datasets as the day of symptom onset for non-severe patients or day of progression for severe patients. A total of 29 lipids were identified in common among the 4 datasets (Fig 5A–D, Table S2). Eight lipids from the PE class [PE(16:0/18:2), PE (16:0/20:4), PE(16:0/22:6), PE(18:0/18:1), PE(18:0/18:2), PE(18:0/20:4), PE(18:0/22:6), PE(18:1/18:2)] and four lipids from PC or PI class of phospholipids [PC(16:0/16:1), PC(16:0/18:1),PC(18:0/18:1), PI(18:0/18:2)] were higher in the non-resolving trauma patients (72h) or severely ill COVID-19 patients in at least one dataset.

We conducted an in-depth comparison between the two trauma datasets to ensure the reproducibility of our findings. A total 75 lipids from 9 sub-classes were found to be in common between PAMPer and TD-2 datasets (Fig S5 A&B). There was remarkable consistency in the relative changes of early drop and late increase in most lipids over time and based on outcome group. The elevated lipids in the non-resolving patients at 72h were almost entirely in the PE, MAG and DAG classed in both the PAMPer (23/26) and TD-2 (18/19) datasets. TAG, LPE, LPC, and DCER were not measured in TD-2 and therefore are not included in this comparison. The consistent findings between the two trauma datasets included a severity-associated drop in all lipid classes early in the clinical course and an increase in lipids, most from the PE and glycerolipid classes between 2–5 days post-injury in patients with a prolonged recovery course.

### Generation and evaluation of a Lipid Reprogramming Score

**Figure 6.**
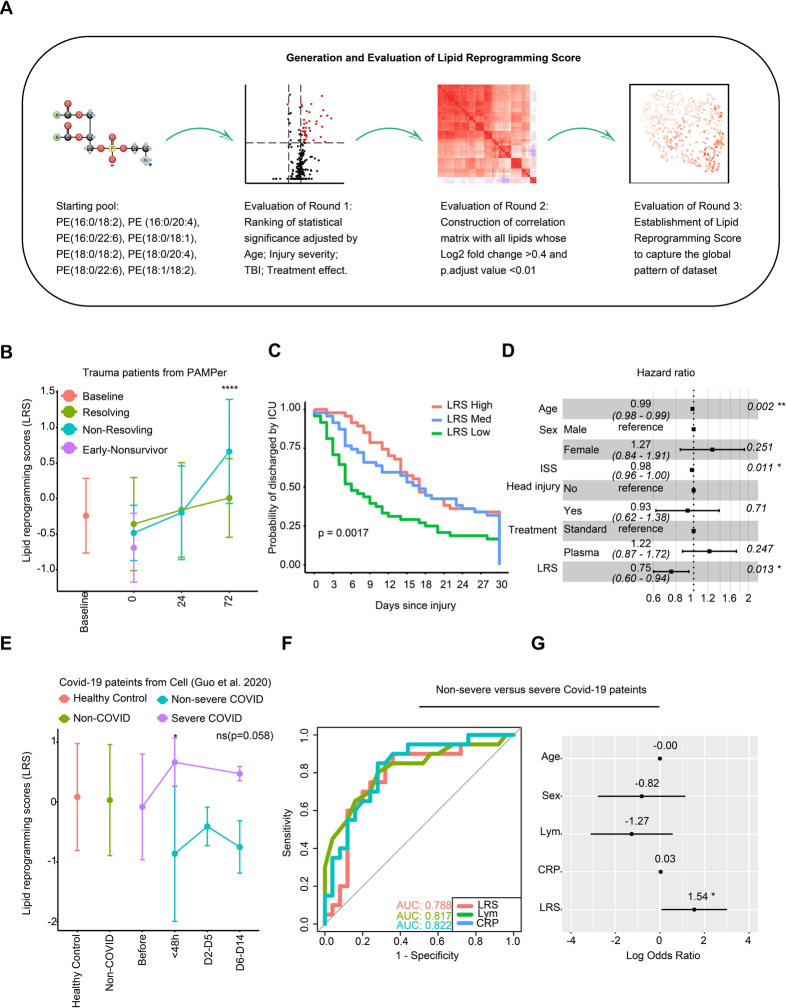
Lipid Reprogramming Score (LRS) is an independent risk factor for outcome after trauma or COVID-19 **(A)** Graphical scheme of generation and evaluation of LRS. **(B)** Comparison of LRS from patients with trauma. Patients are grouped by outcome and sampling timepoint. Center dots and error bars represent median value and median absolute deviation, respectively. **(C)** Recovery probability (defined as discharged from intensive care unit) of different LRS groups across days after injury revealed by K-M curve. LRS groups are based on tertiles at 72h after admission for each patient. **(D)** Forest plot showing hazard ratio of clinical factors and LRS score for recovery using a Cox regression model. **(E)** Comparison of LRS for patients with COVID-19. Patients are grouped with diseases outcome and sampling timepoint. Center dots and error bars represent median value and median absolute deviation, respectively. **(F)** Comparison of predictive value of LRS, lymphocyte count, and CRP for Non-severe versus Severe outcome for the COVID-19 cohort from Guo et al by ROC curve. **(G)** Forest plot showing log odds ratio of clinical factors from logistical regression and LRS score for Non-severe versus Severe COVID-19 patients. Abbreviations: ISS, injury severity score; Lym, lymphocyte count; CRP, C-reaction protein. Asterisks in (B) indicate statistical significance in based on 2-way AVOVA test of time-series analysis of resolving and non-resolving groups. Pairwise Comparisons was conducted by Estimated Marginal Means test. The P value was adjusted by Benjamini-Hochberg method: **** < 0.0001. Asterisks in (E) indicate statistical significance based on Kruskal-wallis test among 6 groups of COVID-19 patients with post-hoc analysis of Dunn test. The P value was adjusted by Benjamini-Hochberg method: *, < 0.05. Asterisks in (D&G) indicate statistical significance in multi-variable regression model: *, < 0.05; **, < 0.01.

To quantify the changes in lipids associated with critical illness in trauma and COVID-19 patients, we used eight PE species common to all four datasets to generate a Lipid Reprogramming Score (LRS) (Fig 6A). Three independent methods were used to define the relationship between the LRS and global lipidomic patterns and outcomes. First, a comparison between non-resolving and resolving trauma patients using logistical regression with Age, ISS, and treatment as co-variables yielded a ranking of lipids detected in PAMPer dataset (Table S3). The eight PE species ranked at ranking at 3, 41, 63, 109, 110, 142, 206, and 294 respectively (Volcano plot shown in Fig S6A). In addition, we found that 27 lipids belonging to TAG class of lipids and 7 additional PE lipids were significantly higher in non-resolving patients at 72h (adjusted p<0.01, log foldchange>0.4). This differential analysis also yielded three LPC that were significantly lower. Next, we constructed a matrix that correlated the initial eight PE in the starting pool with these 37 differentially expressed lipids (Fig S6B). The starting PE were correlated positively with several other PE and 27 TAG, and negatively correlated with the three lower LPC species. This indicates that the eight PE common to all four datasets may also be representative of an overall reprogramming that includes upregulation of TAG release and a suppression of LPC release into the circulation. We generated a LRS represented as a mean z-score for each patient across all three timepoints and plotted them in a UMAP plot (Fig S6C) in order to further reveal their relationships with global lipidome patterns. We found that the gradient in the LRS increased from left-to-right along the x-axis in the UMAP plot, which was consistent with the outcome-based pattern at 72h. We then transformed the score into a categorical variable with three thresholds based on tertiles (Low, Medium, High) for all PAMPer patients surviving at 72h (Fig S6D). When displayed on a UMAP plot, the separation of patients into low, medium, and high LRS tertiles distributed the patients similarly to that seen using the continuous LRS. Thus, both the continuous and categorical LRS values represent the magnitude of global changes in the circulating lipidome and may be useful for correlating the lipidomic changes with other patient features.

### Risk assessment using LRS for patients with trauma or COVID-19

We next investigated whether the LRS was associated with outcomes in trauma or COVID-19 patients. Time-series analysis suggested that non-resolving trauma patients experienced dramatic increases of LRS at 24 to 72h post-trauma compared to resolving patients (Fig 6B). Recovery analysis revealed that LRS-high and LRS-medium groups experienced a longer period prior to recovery than patients in the LRS-low group (Fig 6C). In addition, trauma patients with medium or high LRS were associated with higher injury severity, lower admission blood pressure, mass transfusion, higher INR, and higher incidence of NI and MOF (Table S4). High LRS was also associated with lower probability of recovery (HR:0.75, Cl:0.60–0.94) even when adjusted for age, ISS, TBI, and treatment effect in a Cox regression model (Fig 6D). To validate our finding using a second trauma population, we adopted the same strategy to construct the LRS using TD-2, which was dominated by resolving trauma patients. The time-series analysis, recovery curve, and Cox regression model all showed similar correlations of LRS with outcomes in TD-2 as seen in PAMPer trial patients (sFig 6D, F and G). We then tested whether we could generalize the LRS for the two COVID-19 patient datasets using the same approach. The Shui, et al.^17^ COVID-19 dataset lacked detailed clinical data; therefore, we only compared differences in LRS among the four outcome groups defined by the authors of the study. We found that moderate and severe COVID-19 patients had a higher LRS compared to healthy subjects (Fig S6E). Consistent with these findings, the LRS was also significantly higher in the severe group when compared to the non-severe COVID-19 patients in the dataset of Guo, et al^16^ (Fig 6E). We also observed an upward trend in LRS during the time window preceding progression (< 48h after progression, Fig 6E). C-reactive protein (CRP) and lymphocyte count are known to correlate with worse outcome in COVID-19 patients^24^. We compared LRS with these two variables to classify severe versus non-severe patients. The AUC score for LRS, lymphocyte count, and CRP was 0.788, 0.817, and 0.822, respectively (Fig 6F). Finally, multi-variable logistical regression suggested that LRS is an independent risk factor for COVID-19 patients (Log_2_ OR: 1.54, Fig 6G). Thus, a score based on the levels of a subset of circulating lipids associates with features in trauma and Covid-19 patients that predict a complicated clinical course.

### Association between LRS and systemic markers of inflammation and endothelial dysfunction in trauma patients

**Figure 7.**
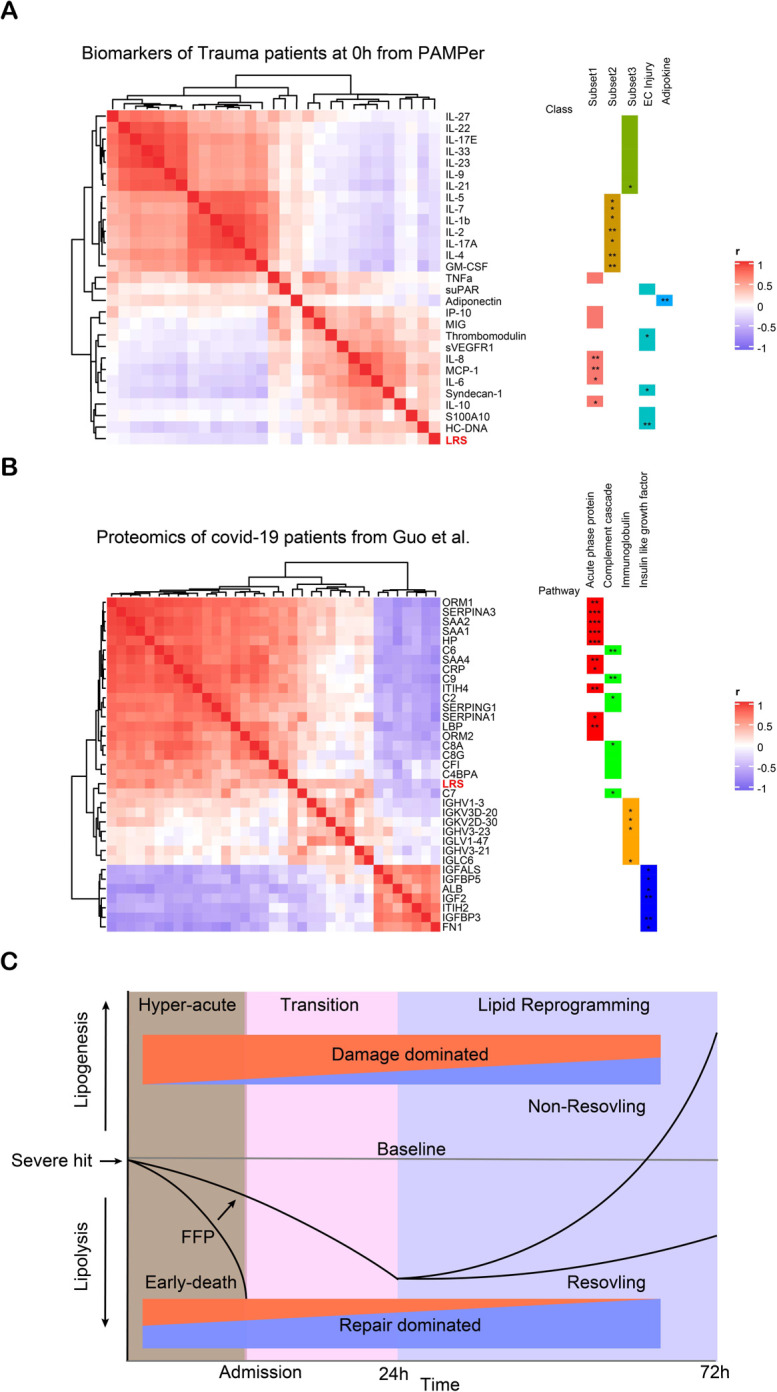
Association between LRS and circulating biomarkers **(A)** Heatmap showing correlation of LRS and circulating biomarkers in 0h upon admission in trauma patients, measured by Spearman correlation coefficients. **(B)** Heatmap showing correlation of LRS and circulating proteins in COVID-19 patients, measured by Spearman correlation coefficients. **(C)** Schematic of proposed paradigm showing the relationship between circulating lipid levels and outcomes after severe injury. Early loss of circulating lipids correlates with adverse outcomes while failure to resolve critical illness is associated with the selective increase in glycerolipids and PE. Asterisks in (A&B) indicate statistical significance for correlation coefficient. P-values are approximated by using the t distributions: *, < 0.05; **, < 0.01; ***, <0.001.

We next sought to determine if the LRS correlated with circulating markers of inflammation or endothelial and tissue damage. A correlation matrix was constructed using data from the 137 PAMPer patients alive at 72h that had complete data for lipids, 21 cytokines and chemokines, endotheliopathy markers, and tissue injury markers across time after injury (Time 0h: Fig 7A, Times 24 and 72h: Fig S7A&B). Across the three time points, LRS correlated positively with various pro-inflammatory Subset 1 cytokines/chemokines, and endotheliopathy and tissue injury biomarkers. Conversely, LRS correlated negatively with subset 2 (lymphocyte-related) and subset 3 (protective/ reparative) cytokines and an adipokine (Adiponectin). These findings suggest that the changes in the circulating lipidome at 72h, represented by an elevated LRS, associates with biological process that drive worse outcomes (e.g. inflammation, endotheliopathy, and tissue injury).

### Association between LRS and the proteome for COVID-19 patients

To further identify possible factors or pathways contributing to a pathologic lipidome signature, we correlated the LRS with circulating proteomic data from the COVID-19 study published by Guo, et al^16^. Using 42 subjects with both metabolomics and proteomics data, we identified 150 proteins that correlated positively (spearman correlation coefficient r > 0.3) with the LRS (Fig S7C). Pathway enrichment analysis revealed that the LRS was associated with neutrophil degranulation, platelet degranulation, and the complement cascade (Fig S7C and Fig S7E). Negatively correlated (spearman correlation coefficient r < −0.3) proteins (n=24) were enriched in regulation of insulin-like growth factor-1 (IGF-1) transport and uptake, and post-translational protein phosphorylation (Fig S7D and Fig S7F). To further seek biological significance, we selected 40 representative proteins from the positive and negative correlating groups to construct a correlation matrix (Fig 7B). Components of the LRS were clustered in the module comprised acute phase proteins, the complement cascade, and immunoglobins and were correlated negatively with modules associated with IGF-1. Our findings using data from COVID-19 patients suggests that excessive acute phase and immune responses and impaired metabolism associates with a pathologic circulating lipid signature across several causes of acute critical illness.

## Discussion

The main goal of this study was to correlate the temporal patterns in the circulating lipidome with outcomes in the early evolution of critical illness in humans. Using trauma as a model, we found that three distinct clinical trajectories each align with comprehensive changes in the patterns of circulating lipids. These relationships are depicted in a summary diagram in Fig 7C. The findings include: (1) A dramatic drop in all classes of lipids in the hyperacute phase after of severe injury that was most extreme in patients destined to die. Early FFP mitigated this rapid drop in lipid levels and was associated with improved outcomes; (2) A persistent lowering of circulating lipids through 72h in patients that resolved their critical illness early; (3) A delayed rise in circulating in DAG, TAG, and PE species in patients that went on to experience persistent critical illness. Remarkably, the over-representation of PE species in trauma patients with critical illness was easily identified in critically ill patients in a validation trauma dataset and two COVID-19 datasets. A Lipid Reprogramming Score derived from PE was an independent risk factor for worse outcome and correlated with excessive proinflammatory and acute phase responses. Although there have been multiple metabolomics studies characterizing the circulating metabolome in critical illness^12,16,17,25,26^, to date there are no reports focusing on the comprehensive temporal lipidome changes in this disease context. We show that lipids may be sensitive markers of the host response to systemic stress and serve as prognostic biomarkers of critical illness.

Among the most pronounced changes observed in our study was the early loss of all classes of lipids in the circulation after injury. A study of 32 trauma patients showed that blood triglyceride levels were significantly lower in 9 non-survivors within 28 minutes of injury, suggesting that injury-induced decreases in circulating lipids may begin very early after a severe trauma^27^. Our healthy controls were non-fasting and sampled throughout the day to align with the presentation of the typical trauma patient. Therefore, the differences between controls and injured at time 0h are unlikely to be due to dietary effects. While the degree of the decline in lipids associated with clinical outcomes, the incidence was not dependent on injury severity. A stress hormone-induced hypermetabolic state with associated increased catabolism is seen after trauma and other causes of critical illness^6,28^ and may explain the persistent decline in circulating lipids. The catabolism response generates energy substrates from carbohydrates, fats, and protein in an “all or none” manner that, like our findings, is not influenced by injury severity^29^. It is reasonable to speculate that the abrupt loss of lipids may be due, in part, to the uptake and catabolism of lipids to meet the energy demands. The finding that patients that die within first 72h experience the greatest magnitude in lipid loss from the circulation raises the interesting possibility that a circulating energy substrate crisis contributes to the early mortality.

Administration of FFP in route to the trauma center improves early survival and we show here that this also results in higher levels of circulating lipids. This was especially true for glycolipids, including TAG, DAG, and MAG, which are rich energy substrates. In addition to providing a source of lipids, FFP also contains proteins involved in coagulation, and many other factors likely to contribute to its salutary actions. FFP is well known to reduce bleeding complications and we have recently reported an association of FFP administration with a prevention of endothelial dysfunction and an excessive inflammatory response^19,30^. The correlative changes in early lipid levels and outcomes in our study point to lipids as another potential beneficial component of FFP.

In stark contrast to the early changes in circulating lipids, a subset of lipids (predominantly TAG, DAG, and PE) began to rise in the circulation between 24 and 72h in patients that subsequently exhibited a slow recovery or die. In addition to lipolysis and hypermetabolism, patients with critical illness experience pathologic alterations in liver such as hepatic steatosis^31–35^. Studies in severe burn trauma associate the browning of white adipose tissue with enhanced lipogenesis in liver^36,37^. Interestingly, the inter-class correlation network among the lipids we identified at 72h is similar to the lipogenesis pathway in the liver. This suggests that the liver is one of the sources of the glycolipids and PE that appear in the circulation and that these reflect ongoing systemic inflammation and metabolic stress. That DAG, TAG, and PE are linked though a common synthesis pathway further supports this possibility^38^. Several specific lipid species [e.g. PC(16:0/18:1), PC(18:0/18:1)] contribute to inter-organ (liver, muscle and adipose tissue) communication^39^. We observed that PC (16:0/18:1) and PC (18:0/18:1) were higher at 72h in the non-resolving trauma patients or severe Covid-19 patients, raising the possibility for a lipid reprogramming process across organs during persistent critical illness.

We derived a LRS that reflects the magnitude of lipid reprogramming associated with delayed adverse outcomes. We found that higher LRS at 72h is an independent risk factor for recovery. Higher LRS was also observed in the sickest COVID-19 patents and even preceded the onset of critical illness. This indicates that lipid reprogramming involving higher levels of a subset of PE in the circulation is a feature common to multiple etiologies of critical illness and that PE might be useful as a biomarker of a pathologic host response. Noticeably, only TAG and DAG comprised of unsaturated fatty acids increased in non-resolving patients. These fatty acids include Eicosapentaenoic Acid (EPA) and Docosahexaenoic Acid (DHA), which are precursors for lipid mediators involved in inflammation resolution and tissue repair^11,40,41^. Thus, in addition to providing a source of lipids for systemic energy needs through the release of acyl glycerides, this response might reflect the host’s attempt to resolve the ongoing inflammatory response and tissue injury.

Global lipid metabolism is regulated by many factors such as pro-inflammatory mediators, adrenergic stress, and regulatory hormones^11,32,36,42,43^. Propranolol or IL-6 receptor blockade can attenuate the browning of white adipose tissue and hepatic steatosis in experimental burn trauma^36^. Interestingly, we also found that the LRS is positively associated with the pro-inflammatory response, the acute phase response, endothelial injury, and coagulation but inversely correlated with mediators shown to contribute to tissue protection and repair. This relationship persisted throughout the 72h observation period. IGF-1 and adiponectin are produced by liver and adipose tissue, respectively, and are functionally associated^44^. Both hormones enhance fatty acid oxidation as an energy source and were negatively correlated with the LRS, consistent with a dysregulated lipid reprogramming in patients with persistent critical illness.

Our study has several limitations. Many of the observations are correlative and prospective validation will be required to establish the value of the LRS as a prognostic tool. The mechanistic relationship between the changes in lipids in the circulation do not necessarily reflect lipid metabolism within specific organs or tissues. Finally, the functional contributions of the observed lipid changes to patient outcomes remain to be established in patients.

In conclusion, our findings provide a new paradigm for the lipid response to a severe and acute systemic stress leading to critical illness (summarized in Figure 7C). Our causal modeling and correlation analyses place lipolysis a central regulator of the evolution from acute disease onset to critical illness in humans. The features of lipogenesis we identified appear to be common to critical illness due to multiple etiologies and potentially useful for predictive modeling and target identification. Both the proposed new paradigm and our comprehensive datasets will be useful for further study of altered lipid metabolism in acute critical illness.

## Methods

### Study population and samples

We conducted longitudinal sampling of plasma (0h; 24h; 72h after admission) from 193 patients with trauma prospectively enrolled in the PAMPer trail,^19^ along with 17 healthy subjects. The detailed workflow is shown in Fig S1. The primary aim of PAMPer trail was to test if administering prehospital fresh frozen plasma (FFP) during air medical transport can reduce in-hospital mortality for severely injured trauma patients. Values for clinical and physiological variables with biomarkers of injury and inflammation given in the manuscript were reported from previous studies^19,30^. The outcome of trauma patients was defined as: Resolving (Survival with ICU stay < 7 days); Non-resolving (Survival with ICU stay >= 7 days or non-survival with death day >3 days) and Early-nonsurvivors (Non-survival with death day <=3 days). Blood samples were collected using vacuum isolation tubes with anticoagulant of Heparin sodium, which were centrifuged at 4°C and plasma fractions were stored at −80°C for further analysis.

This study was approved by the IRB of University of Pittsburgh as previously described^19^. The Emergency Exception from Informed Consent (EFIC) protocol from the Human Research Protection Office of the US Army Medical Research and Material Command was applied to this study. Registered information and detailed study protocol are available on https://clinicaltrials.gov/ct2/show/NCT01818427.

### Targeted lipidomics by LC-MS/MS

Samples were shipped to Metabolon (Durham, NC, USA, www.metabolon.com) for complex lipid panel processing. Lipids were extracted from the plasma in the presence of deuterated internal standards using an automated BUME extraction according to the method of Lofgren et al^45^. The extracts were dried under nitrogen and reconstituted in a dichloromethane: methanol solution containing ammonium acetate. The extracts were transferred to vials for infusion-MS analysis, performed on a Shimadzu LC with nano PEEK tubing and the Sciex SelexIon-5500 QTRAP. The samples were analyzed via both positive and negative mode electrospray. The 5500 QTRAP was operated in MRM mode with a total of more than 1,100 MRMs. Individual lipid species were quantified by taking the ratio of the signal intensity of each target compound to that of its assigned internal standard, then multiplying by the concentration of internal standard added to the sample. Lipid species concentrations were background-subtracted using the concentrations detected in process blanks (water extracts) and run day normalized. The internal standard serve as technique replicate was run multiples times throughout the experiment. Instrument variability was evaluated by calculating median relative SD (RSD) from the quality control sample matrix.

### Lipidomic data pre-process and dimension reduction

Lipids were named according to its sub-class and fatty acid composition; (e.g. PE (16:0/18:2) means this lipid belongs to phosphatidylethanolamine (PE) class and it was synthesized from palmitic acid (C16:0) and linoleic acid (C18:2)). Lipid with over 80% missing quantitative values were discarded due to the concern of low quality. Other missing values for each lipid species were imputed with the minimum concentration. Lipid class concentrations were calculated from the sum of all molecular species within a class, and fatty acid compositions were determined by calculating the proportion of each class comprised by individual fatty acids.

Normality of each lipid species distribution was tested by Shapiro-Wilk test and Q-Q plot. No transformation was conducted because most lipid species obey normal distribution or was near normal distribution. A two steps approach of dimension reduction from both linear and non-linear methods were applied. Principle Component Analysis (PCA) was performed on z-score scaled concentration of each lipid species. Then, Uniform Manifold Approximation and Projection (UMAP) was conducted by using the first 20 PCs. All subjects grouped by outcome or timepoint were visualized in UMAP plot. No obvious outliers were identified in the UMAP plot.

### Casual inference analysis

Casual inference was performed by using the on-line CausalMGM^46^ and the command-line tool for FCI^47^. Early death (death day <= 3 after admission) was set as the outcome and all other variables which may be related to early death were kept as input (Clinical information: Age; Trauma brain injury (TBI), Injury severity (ISS); GCS; TRISS, Hemostasis: INR; Coagulopathy. Intervention: Prehospital fresh frozen plasma (FFP); Prehospital transfusion volume of crystalloid; Prehospital intubation; Transfusion volume in first 24h after admission, Biomarkers: 21 cytokines with 7 endothelial injury related markers, total lipid concentration). Continuous variables of biomarkers were log2 transformed and z-score scaled to meet the assumption of normality. Categorical variables were tested to meet the assumption of multi nominal distribution. To select the optimal α-value threshold for the conditional independence tests of the FCI we used a nested leave-one-out cross validation. In each round, directed graphs were learned from all but one samples at different α-values (α = {0.01, 0.05, 0.1, 0.15, 0.2, 0.25}). The variables in the Markov blanket of the “Early death” variable (i.e., parents, children and spouses) in each α-value were used to train a logistic regression model. This model was then used to predict the “Early death” in the left-out sample. The procedure was repeated for all samples and Receiver Operator Characteristic (ROC) curves were constructed for each α-value. The value of α = 0.2 produced models with the best Area Under the ROC Curve (AUC=0.80). The final causal network presented in Fig. 4G was constructed on the full dataset using the α = 0.2 for the conditional independence tests.

### Correlation network and lipid biosynthesis pathway

Correlation networks were constructed using 412 lipids based on a Pearson correlation coefficient matrix from all samples. All lipids in the class of MAG; CE; PI; LPE; LPC; SM; CER; LCER; HCER; DCER were kept. Lipids of TAG; DAG; PE; PC were kept at top 100; 30; 40;40 variable species respectively to reduce the complexity of network. Variance Stabilizing Transformation (vst) method was used for identifying variable lipids and mean-var plot for each class was examined to ensure the stability. The threshold of the correlation coefficient was tuned from 0.5 to 0.8 and then set at 0.7 based on the following considerations: 1. Balance between intra-class correlation and inter-class correlation; 2. Preference for a higher threshold to reduce false positive relationships. Cytoscape (version 3.8.0) was used to construct the inter-class and intra-class network and layout was set as circular^48^. Lipid biosynthesis pathways were summarized from previous published literature.

### Establishment and application of lipid reprogramming score (LRS)

The main purpose for generation of LRS is to quantitatively measure the magnitude of lipogenesis via several lipid species detected among all datasets. Only species from PE were kept as starting pool because it is the only lipid class identified common to both metabolomic and lipidomic datasets. Approach similar to construct signature score was adopted to generate LRS. Briefly, 8 common PEs (PE(16:0/18:2), PE (16:0/20:4)), PE(16:0/22:6), PE(18:0/18:1), PE(18:0/18:2), PE(18:0/20:4), PE(18:0/22:6), PE(18:1/18:2) were scaled by z-score among patients or health subjects. No feature selection was performed at this step due to the balance of performance and stability. Then, LRS was set as mean value of z-score of 8 PEs. Trauma patients in PAMPer trial who survived at 72h after admission were classified to 3 groups (High, Medium, Low) according to the tertiles of LRS across all patients. LRS was calculated for both trauma and COVID-19 patients and healthy subjects when applied in time-series or comparison analysis. LRS was only calculated for patients with trauma or COVID-19 when applied in multi-variable model of cox regression or logistical regression.

### Recovery analysis

A Kaplan–Meier Curve was used in the recovery analysis for trauma patients from PAMPer or the TD-2 dataset. ICU length of stay was used to estimate the time to recovery for patients due to lack of detailed variables for dynamically monitoring organ dysfunction since injury. Patients who experience early death were excluded for recovery analysis. The ICU length of stay for patients died over 3 days after admission was consider as maximum days in this dataset, because they cannot recover from injury. Patients who experience ICU length of stay over 30 days was consider as censor at day 30.

### Multi-variable regression analysis

Multi-variable model of logistical regression was used for testing the categorical outcome like survival or severity. Only main effect of each factor was evaluated. Demographic information (e.g. age, sex), TBI, TRISS, treatment arm and total lipid concentration at 0h upon admission were included in the logistical regression model for early death in PAMPer dataset. Demographic information (e.g. age, sex), Lymphocyte count, CRP, LRS across each patient were included in the logistical regression for modeling severe COVID-19 patients in dataset of Guo et al. A multi-variable model of Cox regression was used for testing the time to discharged by ICU for trauma patients. Demographic information (e.g. age, sex), TBI, ISS, treatment arm and LRS score among patients at 72h after admission were included in the Cox regression for modeling Non-resolving patients in PAMPer dataset. External validation by using same variables except for treatment arm was conducted in TD-2 dataset.

### Correlation analysis

Two types of correlation analysis either for between two continuous variables or categorial variables and continuous variables were including in this study. Continuous variables like cytokines, biomarkers and total lipid concentration was log2 transformed. Categorial variables like early death, treatment arm, TBI and coagulopathy were transformed into dummy variables. Euclidean distance matrix was calculated for correlation analysis. Spearman correlation coefficient was used for correlation between biomarkers and total lipid concentration or LRS due to consideration of non-linear relationship. Pearson correlation coefficient was used for correlation between lipid species due to the well-identified linear relationship. Statistical analysis for correlation coefficient is conducted by function rcorr() implemented in R package Hmisc(version 4.4.1). P values are approximate by using t distributions.

### Pathway analysis

R package clusterprofiler (version 3.11) was used to conduct pathway analysis for proteins which were correlated to LRS^49^. First, names of 152 positively (spearman r >0.3) and 24 negatively (Spearman r < −0.3) correlated proteins were transformed into Entrez ID. Then, the Reactome database was used to enrich positively or negatively correlated pathways. The P value of enriched terms was adjusted by the Benjamini-Hochberg method. Only pathways that meet a P value < 0.05 was consider to be significant.

### Statistical analysis and visualization

Statistical analysis in this study was performed by using R language (version 3.6.0, https://www.R-project.org/)^50^. Pearson’s χ2 test and Kruskal-Wallis test were used for categorical variables or continuous variables in the contingency table of clinical data. Kruskal-Wallis test with post-hoc analysis by Dunn test was used for multiple group comparisons. Two-way ANOVA with pair-wise comparisons by Estimated Marginal Means test was applied for time-series analysis. P value was adjusted by Benjamini-Hochberg method with less than 0.05 for establishing significance. Visualization of heatmap was performed by using R package Complexheatmap (version 2.5.1)^51^. Hierarchical clustering based on Euclidean distance was applied in rows or columns for heatmap construction.

### External metabolomics or lipidomics dataset

Three external datasets of untargeted metabolomics or lipidomics were included in this study. The first dataset was from survival cohort which consisted of trauma patients with untargeted metabolome measurement^23^. The same criterion for outcome classification was applied in this group of patients to that used for the PAMPer dataset (Resolving: ICU Days <7; Non-resolving: ICU Days >=7). The second dataset was from a cohort of COVID-19 patients with both untargeted metabolome and proteome measurements^16^. The patients were grouped by severity defined in the previous study and days to timepoint 0, which was set as day of progression for severe patients and day of symptom onset for non-severe patients. The third dataset was from separate cohort of COVID-19 patients with both targeted and untargeted metabolome measurements^17^. The patients were not grouped by sampling timepoint because of limited clinical information. Common lipids were identified by unique molecular formula or HMID from Human Metabolome Database among these 3 datasets and PAMPer lipidomic dataset. Mean z-score scaled value for each group for patients or healthy subjects was used to compare the lipid levels among 4 datasets.

## Supplementary Material

Supplement

Supplement

1

## Figures and Tables

**Table 1:** Demographic characteristics of the patients by outcome

Variables	Resolving (N=41)	Non-resolving (N=101)	Early-Nonsurvivors (N=51)	p-value
**Demographics**				
Age (Median [IQR])	48 (± 34)	46 (± 37)	46 (± 42)	0.916
Sex (% Male)	31 (75.6%)	78 (77.2%)	36 (70.6%)	0.668
Race (% White)	35 (85.4%)	89 (88.1%)	48 (94.1%)	0.365
**Injury characteristics**				
ISS (Median [IQR])	21 (± 10)	30 (± 16)	24 (± 23)	<0.001
Head AIS (Median [IQR])	0(± 3.0)	3.0 (± 2.0)	3.0 (± 4.0)	<0.001
TBI (%)	14 (34.1%)	66 (65.3%)	29 (56.9%)	0.003
GCS (Median [IQR])	14 (± 7.0)	3.0 (± 9.0)	3.0 (± 8.0)	<0.001
SBP<70mmHg (%)	19 (46.3%)	41 (40.6%)	25 (49.0%)	0.580
HR (Median [IQR])	120 (± 16)	120 (± 21)	120 (± 39)	0.218
Injury type (% Blunt)	30 (73.2%)	93 (92.1%)	47 (92.2%)	0.017
**Prehospital**				
Treatment arm				
Standard care (%)	25 (61.0%)	48 (47.5%)	36 (70.6%)	0.021
FFP (%)	16 (39.0%)	53 (52.5%)	15 (29.4%)	
Transport time (Median	39 (± 18)	44 (± 17)	42 (± 18)	0.771
CPR (%)	0 (0%)	3 (2.97%)	5 (9.80%)	0.044
Intubation (%)	13 (31.7%)	65 (64.4%)	40 (78.4%)	<0.001
Blood (%)	11 (26.8%)	32 (31.7%)	22 (43.1%)	0.214
Crystalloid (Median	800 (± 1400)	830 (± 1300)	1000 (± 1600)	0.891
PRBC (Median [IQR])	0 (± 1.0)	0 (± 1.0)	0 (± 2.0)	0.233
**Hospital**				
Transfusion 24h (Median	2.0 (± 8.0)	7.0 (± 14)	12 (± 20)	<0.001
PRBC 24h (Median [IQR])	2.0 (± 5.0)	5.0 (± 7.0)	8.0 (± 10)	<0.001
Plasma 24h (Median	0 (± 0)	2.0 (± 4.0)	4.0 (± 8.0)	<0.001
Platelets 24h (Median	0 (± 0)	0 (± 1.0)	1.0 (± 2.0)	0.002
Crystalloid 24h (Median	4800 (± 3800)	5300 (± 4000)	4600 (± 3000)	0.095
Vasopressors 24h (%)	19 (46.3%)	68 (67.3%)	44 (86.3%)	<0.001
INR (Median [IQR])	1.2 (± 0.20)	1.3 (± 0.36)	1.6 (± 0.72)	<0.001
**Other outcomes**				
Coagulopathy (%)	16 (39.0%)	54 (53.5%)	44 (86.3%)	<0.001
ALI (%)	2 (4.88%)	47 (46.5%)	3 (5.88%)	<0.001
NI (%)	3 (7.32%)	43 (42.6%)	\	<0.001
MOF (%)	31 (75.6%)	98 (97.0%)	\	<0.001
Vent days (Median [IQR])	2.0 (± 3.0)	10 (± 8.0)	1.0 (± 0)	<0.001
ICU LOS (Median [IQR])	4.0 (± 3.0)	13 (± 9.0)	1.0 (± 1.5)	<0.001
Hospital LOS (Median	9.0 (± 10)	19 (± 19)	1.0 (± 1.0)	<0.001

Pearson’s χ2 test was used for calculating p value of categorical variables. Kruskal-Wallis test was used for calculating p value of continuous variables. ISS, injury severity score; AIS, abbreviated injury score; TBI, traumatic brain injury; GCS, Glasgow coma score; SBP, systolic blood pressure; HR, heart rate; FFP, fresh frozen plasma; CPR, cardiopulmonary resuscitation; PRBC, packed red blood cells; INR, international normalized ratio; ALI, acute lung injury; NI, nosocomial infection; MOF, multiple organ failure; ICU, intensive care unit; LOS, length of stay.
