# Supplementary material for: Lipidomic Signatures Align with Inflammatory Patterns and Outcomes in Critical Illness
Source: Res Sq. 2021 Jan 8:rs.3.rs-106579. Preprint. [Version 1] doi: 10.21203/rs.3.rs-106579/v1 (PMC7805459; doi:10.21203/rs.3.rs-106579/v1)
Supplement: Supplement [file fb8af2e913bb2fb2154e78d4.docx]

**Supplementary Acknowledgement:**

**The PAMPer study group Investigators and Collaborators**

**University of Pittsburgh, Presbyterian Hospital; Clinical Coordinating center and enrolling site**:

Jason L. Sperry, MD, MPH, Francis X. Guyette, MD, MPH, Mazen S. Zenati, MD, PhD, Joshua B.Brown, MD, MSc, Mark H. Yazer, MD, Darrell J. Triulzi, MD Barbara J. EarlyYoung, BSN, Peter W.Adams, BS, Louis H. Alarcon, MD, Clifton W. Callaway, MD, PhD, Brian S. Zuckerbraun, MD, Matthew D. Neal, MD, Raquel M. Forsythe, MD, Timothy R. Billiar, MD, Donald M. Yealy, MD, Andrew B. Peitzman, MD, Meghan L. Buck, Ashley M. Ryman, Elizabeth A. Gimbel, Erin G. Gilchrist, Meghan Buhay, Chung-Chou H. Chang, Victor B. Talisa, Tianyuan Xu, Multidisciplinary Acute Care Research Organization (MACRO)-MACRO Research Specialists, MACRO Clinical Trials Research Associates.

**University of Tennessee Health Science Center**: Brian J. Daily, MD, Kyle Kalloway, Andrew Yates, Susan Rawn.

**Vanderbilt University Medical Center**: Richard S. Miller, MD, Judith M.Jenkins.

**University of Louisville**: Brain G. Harbrecht, MD, Laura S. Trachtenberg, Randi K. Eden.

**MetroHealth Medical Center/Case Western Reserve University**: Jeffrey A. Claridge, MD, Joanne Fraifogl, Craig Bates, MD.

**University of Texas Southwestern/Parkland Memorial Hospital**: Herb A.Phelan, MD, Christina Howard, Cari Stebbins.

**Texas Health Harris Methodist Hospital**: William R.Witham, MD, Cathy McNeill.

**University of Pittsburgh Medical Center, Altoona Hospital**: A. Tyler. Putnam, MD, Amy Snyder, Jason Ropp.

**John Peter Smith Health Network**: Therese M. Duane, MD,Celeste Caliman, Mieshia Beamon.
