# Supplementary material for: Lipidomic Signatures Align with Inflammatory Patterns and Outcomes in Critical Illness
Source: Res Sq. 2021 Jan 8:rs.3.rs-106579. Preprint. [Version 1] doi: 10.21203/rs.3.rs-106579/v1 (PMC7805459; doi:10.21203/rs.3.rs-106579/v1)
Supplement: 1 [file NIHPPrs106579v1-supplement-1.pdf]

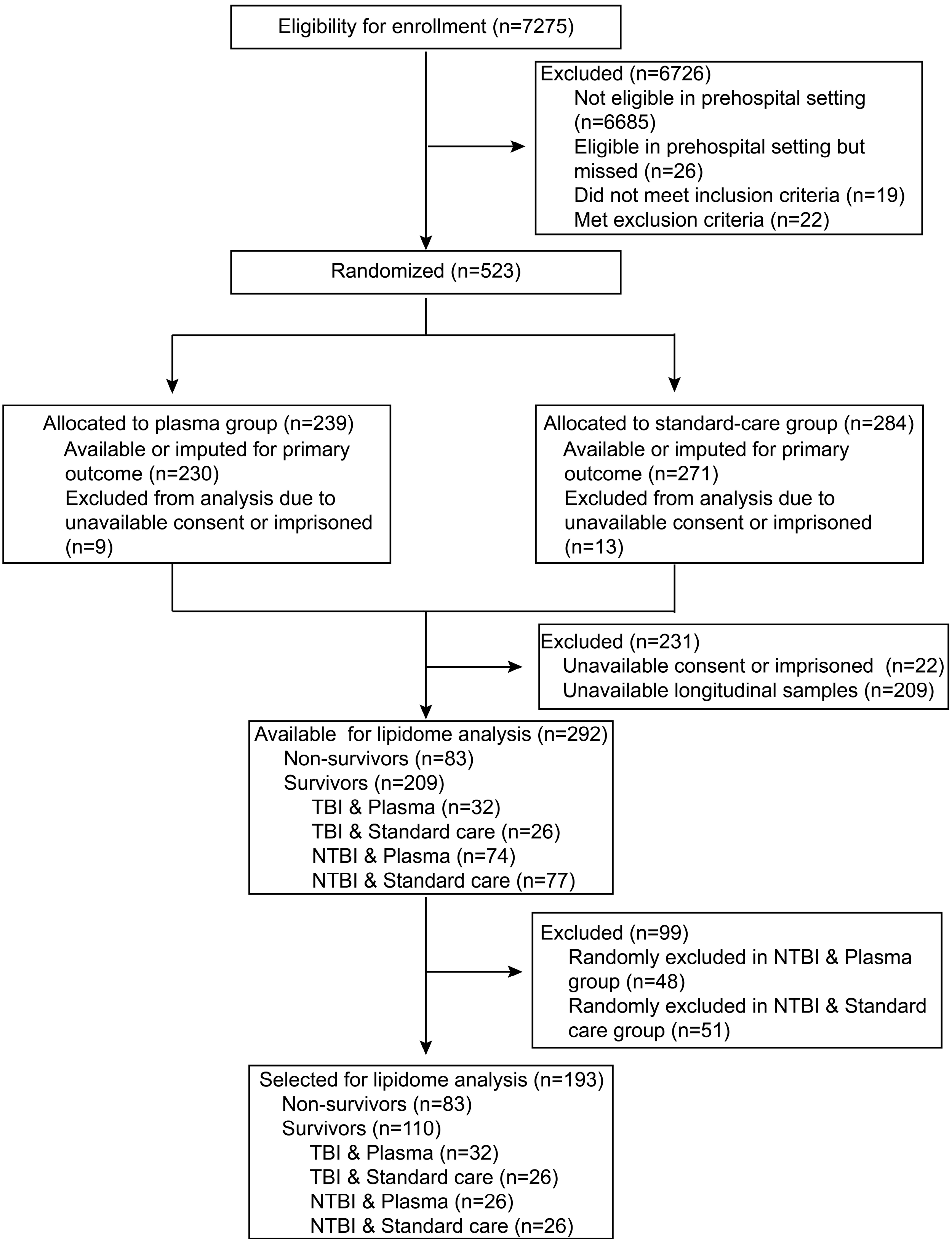

Figure S1. Consort diagram. Screening, randomization and sampling for lipidomic analysis.

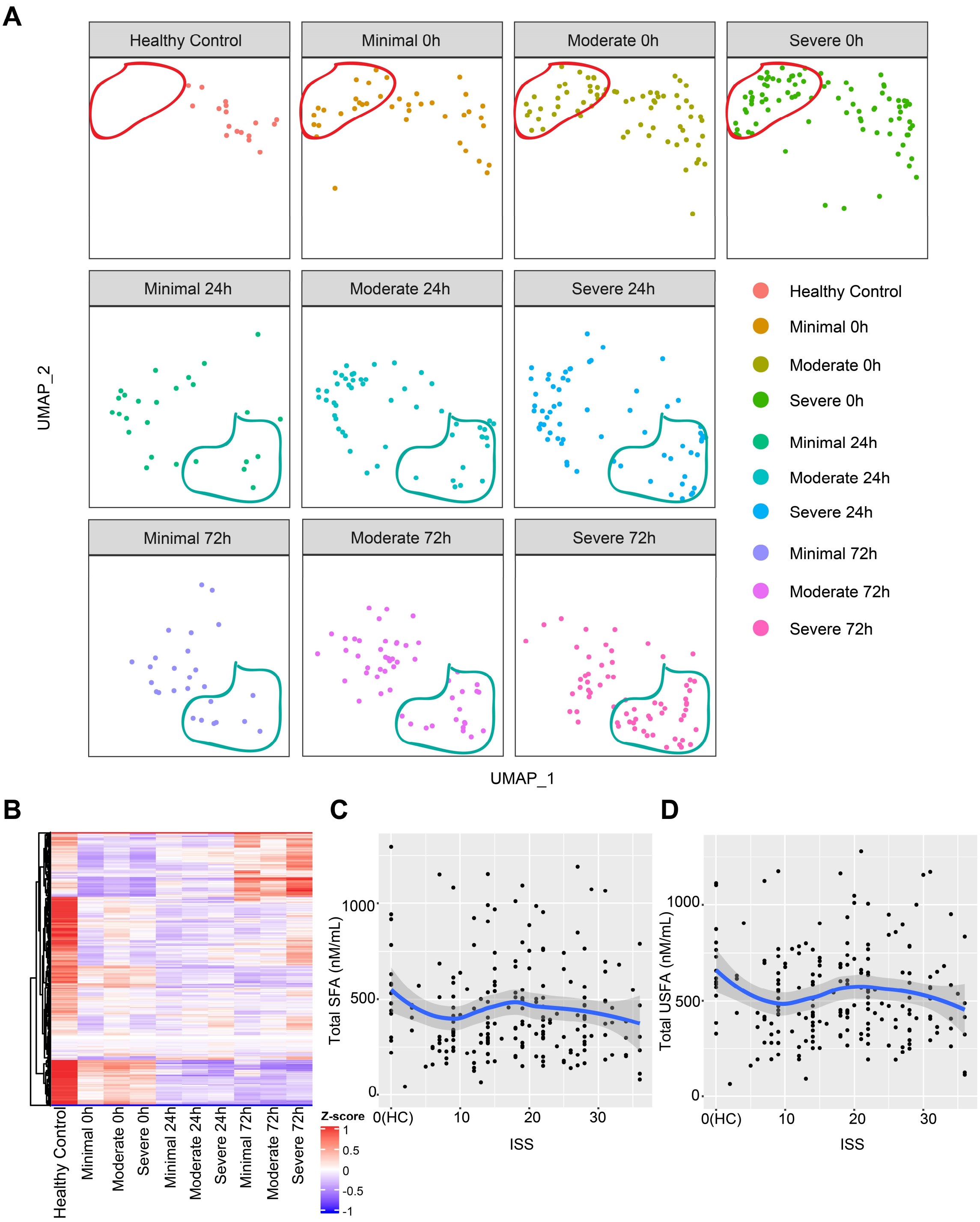

Figure S2. Relationship of the circulating lipidome to injury severity.

(A) Uniform Manifold Approximation and Projection (UMAP) plot shows the distribution of healthy subjects (n=17) and patients with trauma (n=193), grouped by injury severity and sampling timepoints**.** (Minimal: ISS<10, Moderate: 10<=ISS<25, Severe: ISS>=25)

(B) Heatmap showing relative levels of 996 lipid species for healthy subjects and trauma patients, grouped by injury severity and sampling timepoints. Exp, z-score normalized concentration. Rows are clustered by hierarchical clustering.

(C-D) Relationship of ISS to absolute concentration of total saturated fatty acid (C) and unsaturated fatty acid (D) at 0h revealed by scatterplot.

ISS, injury severity score; SFA**:** saturated fatty acid; USFA: unsaturated fatty acid.

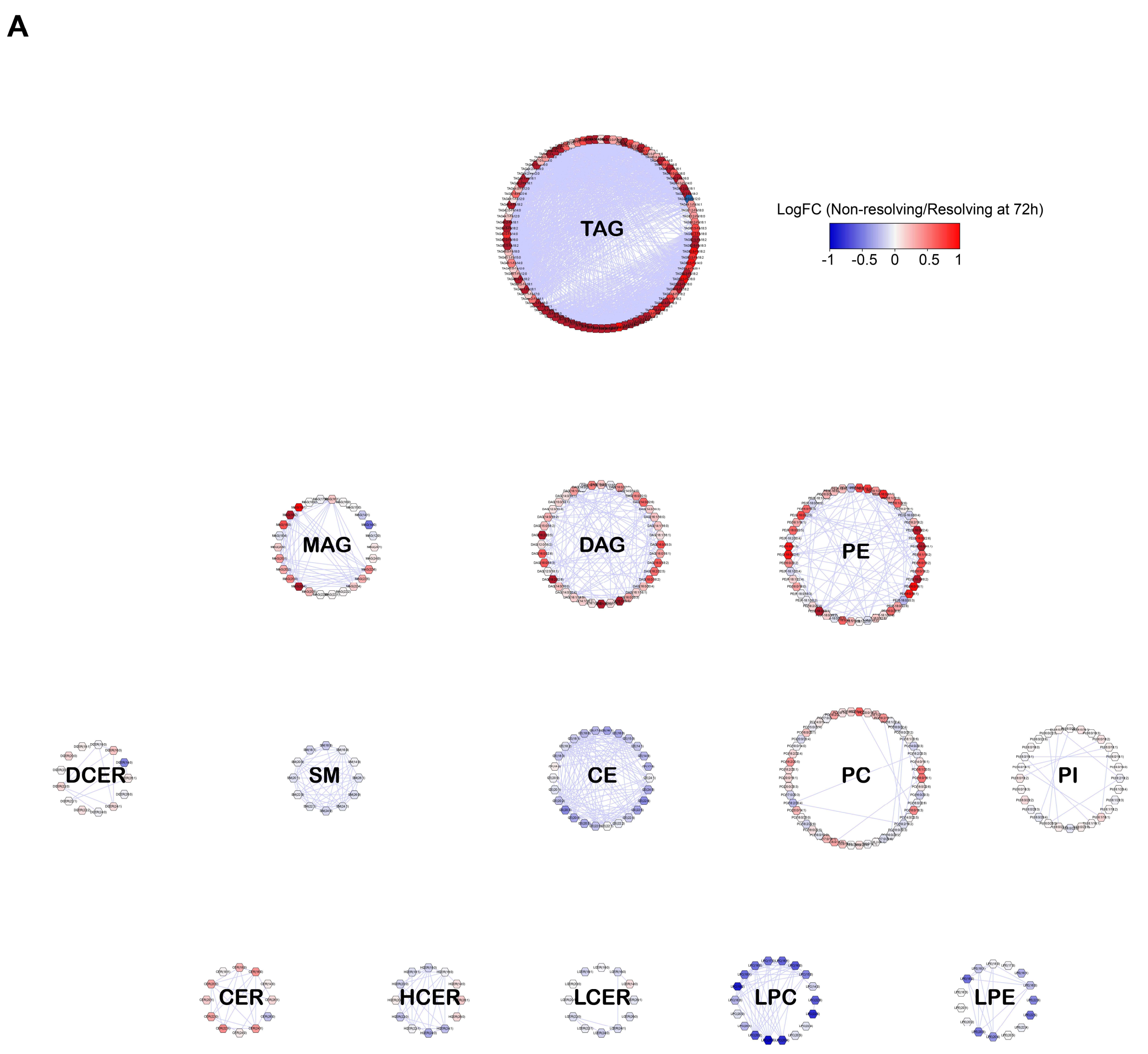

Figure S3. Lipid intra-class network in non-resolving trauma patients at 72h

Correlation network for 412 lipids from 14 classes from the lipidomic dataset. Each dot indicates a lipid and is organized by circle if it belongs to one class. Edge between 2 dots designates high correlation (Pearson coefficient > 0.7). Only intra-class correlations are shown. Coloring indicates levels between non-resolving and resolving trauma patients.

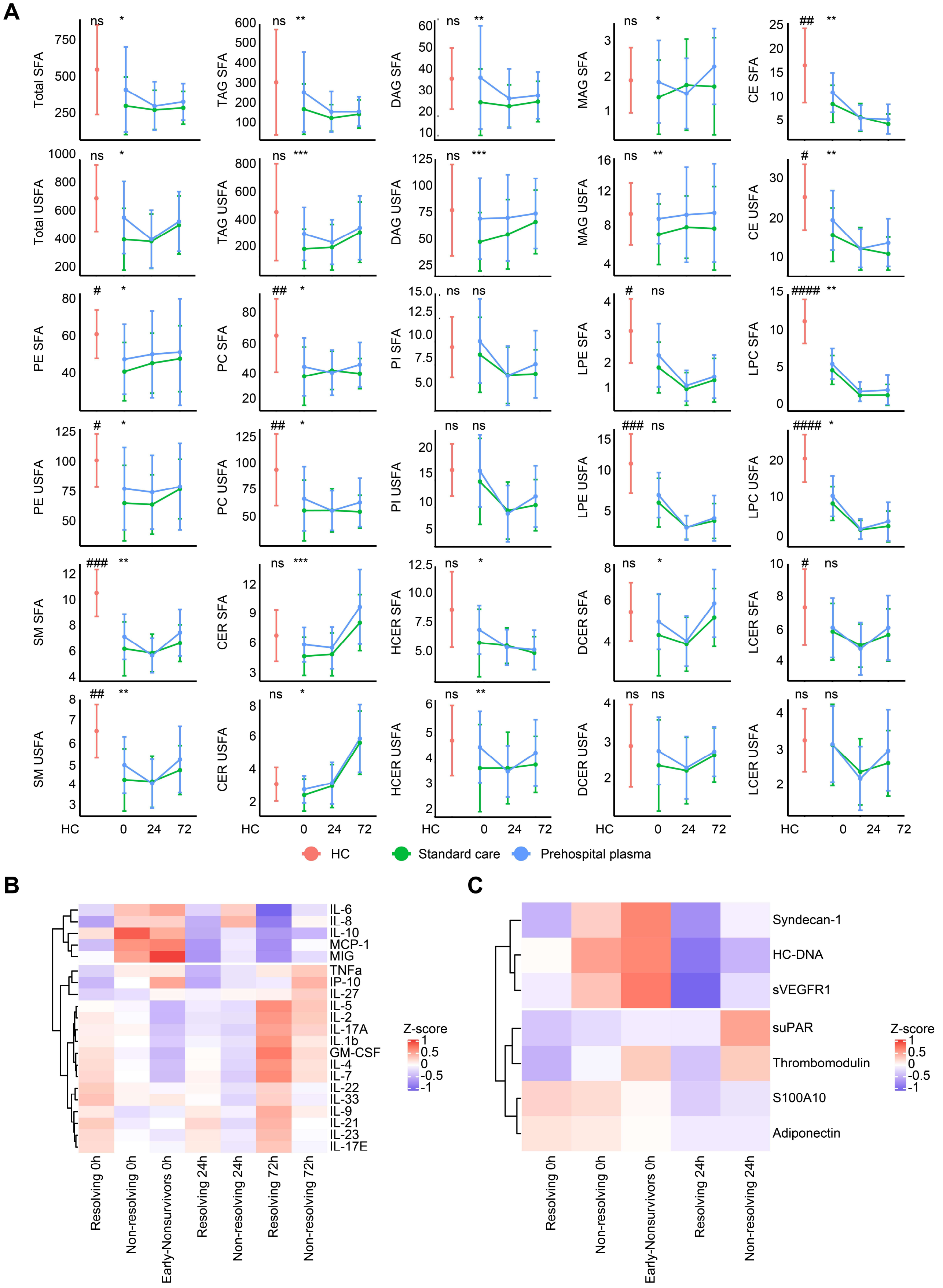
A
Total SFA
TAG SFA
DAG SFA
MAG SFA
CE SFA
Total USFA
TAG USFA
DAG USFA
MAG USFA
CE USFA
PE SFA
PC SFA
PI SFA
LPE SFA
LPC SFA
PE USFA
PC USFA
PI USFA
LPE USFA
LPC USFA
SM SFA
CER SFA
HCER SFA
DCER SFA
LCER SFA
SM USFA
CER USFA
HCER USFA
DCER USFA
LCER USFA
HC 0 24 72
HC
Standard care
Prehospital plasma
B
IL-6
IL-8
IL-10
MCP-1
MIG
TNFa
IP-10
IL-27
IL-5
IL-2
IL-17A
IL.1b
GM-CSF
IL-4
IL-7
IL-22
IL-33
IL-9
IL-21
IL-23
IL-17E
Z-score
Resolving 0h
Non-resolving 0h
Early-Nonsurvivors 0h
Resolving 24h
Non-resolving 24h
Resolving 72h
Non-resolving 72h
C
Syndecan-1
HC-DNA
sVEGFR1
suPAR
Thrombomodulin
S100A10
Adiponectin
Z-score
Resolving 0h
Non-resolving 0h
Early-Nonsurvivors 0h
Resolving 24h
Non-resolving 24h

Figure S4. Prehospital fresh frozen plasma (FFP) can enhance levels of major lipid class

(A) Comparison of circulating total lipid concentration between standard care and prehospital FFP. Lipids are grouped by classes and fatty acid (saturated or unsaturated) contained in the lipids. Patients are grouped by treatment and sampling timepoints. Center dots and error bars represent median value and median absolute deviation, respectively. SFA**:** saturated fatty acid; USFA: unsaturated fatty acid. Asterisks indicate statistical significance between baseline and prehospital FFP arm. Number sign indicates statistical significance between treatment arms in 0h. Kruskal-wallis test was used among baseline and treatment arms at 0h with post-hoc analysis of Dunn test. p value was adjusted by Benjamini-Hochberg method: *, < 0.05; **, < 0.01, ***, < 0.001; #, < 0.05; ##, < 0.01; ###, < 0.001, #### < 0.0001.

(B) Heatmap shows temporal pattern of circulating cytokines in trauma patients at 0h,24h and 72h after admission.

(C) Heatmap shows temporal pattern of circulating biomarkers in trauma patients at 0h and 24h after admission.

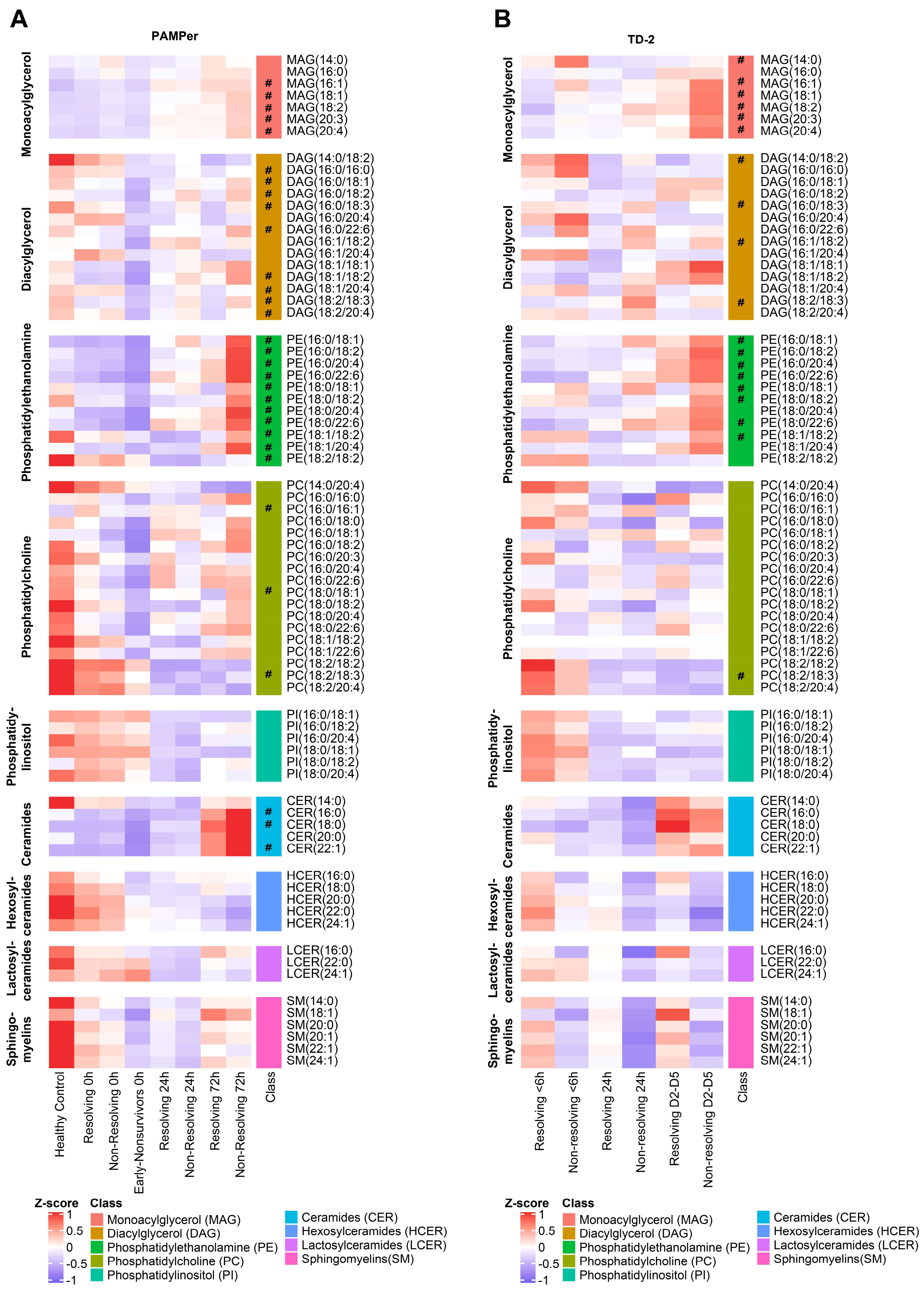

Figure S5. Temporal pattern of common lipids of trauma patients from PAMPer and TD-2.

(A-B) Heatmap shows relative levels of 99 common lipid species from 9 major classes across patients. Patients are group by outcome and sampling timepoint. Data comes from PAMPer lipidomics dataset **(A)** or TD-2 untargeted metabolomics dataset **(B)**.

Number sign (#) indicate lipids with log2 fold change >0.4 between non-resolving and resolving trauma patients at 72h (A); non-resolving and resolving trauma patients at D2-D5 (B).

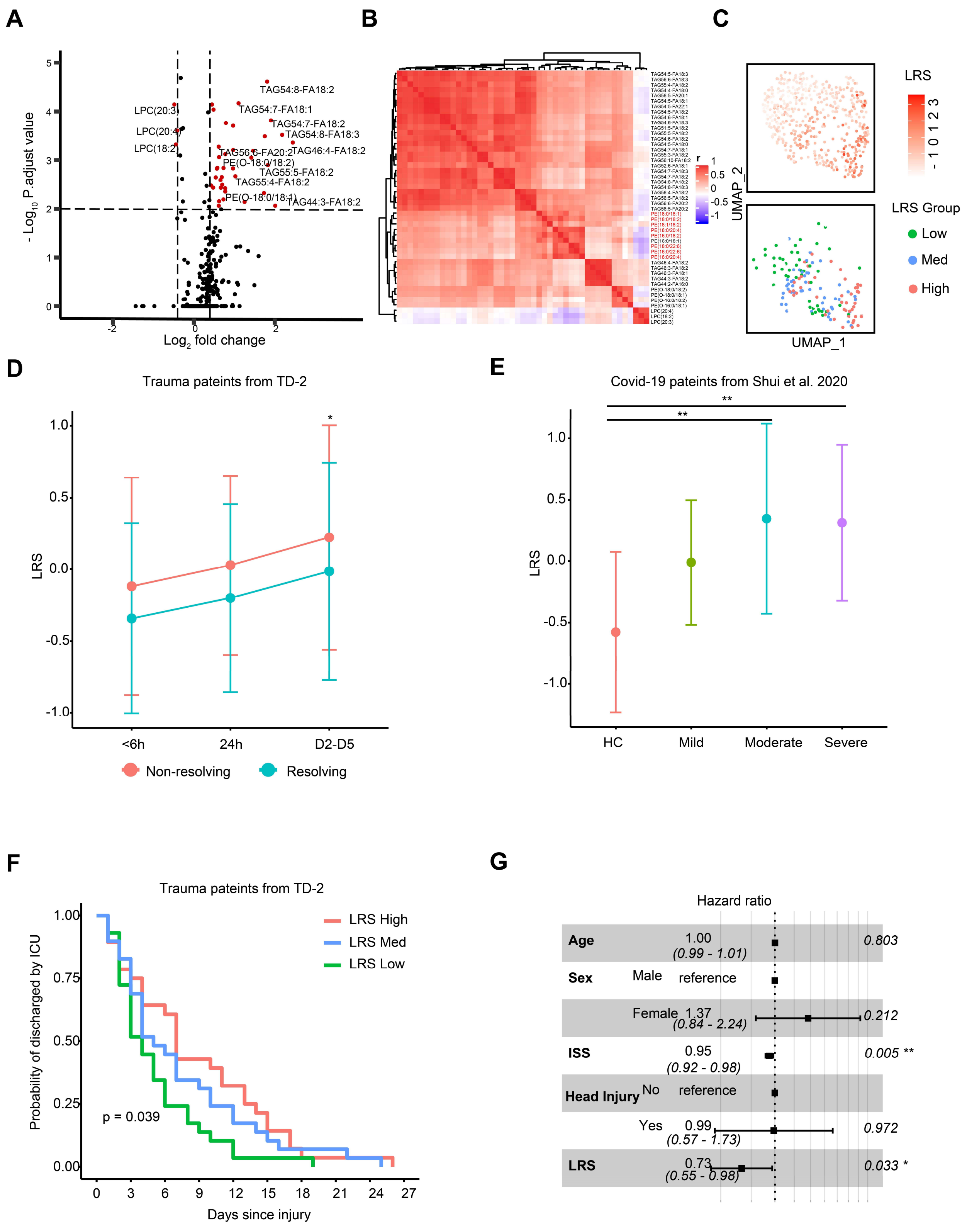

897

898 **Figure S6. Evaluation and external validation of lipid reprogramming score (LRS).**

(A) Volcano plot shows the differential lipids in non-resolving patients compared to resolving patients at 72h after admission.

(B) Correlation heatmap of 8 common lipids and 37 selected differential lipids.

(C) UMAP plot of LRS and LRS group among trauma patients.

(D) Comparison of LRS from patients with trauma in TD-2 dataset. Patients are grouped according to outcome and sampling timepoint. Center dots and error bars represent median value and median absolute deviation respectively.

(E) Comparison of LRS from patients with COVID-19. Patients are grouped with outcome. Center dots and error bars represent median value, median absolute deviation respectively.

(F) Recovery probability (defined as discharged by intensive care unit) of different LRS groups across days since injury revealed by K-M curve in TD-2 dataset.

(G) Forest plot shows the Hazard ratios of clinical factors and LRS score for recovery using cox regression in the TD-2 dataset. ISS, injury severity score.

Asterisks in (D) indicate statistical significance in based on 2-way AVOVA test of time-series analysis of resolving and non-resolving groups. Pairwise Comparisons was conducted by Estimated Marginal Means test. p value was adjusted by Benjamini-Hochberg method: * < 0.05. Asterisks in (E) indicate statistical significance based on Kruskal-wallis test among 4 group with post-hoc analysis of Dunn test. p value was adjusted by Benjamini-Hochberg method: **, < 0.01.

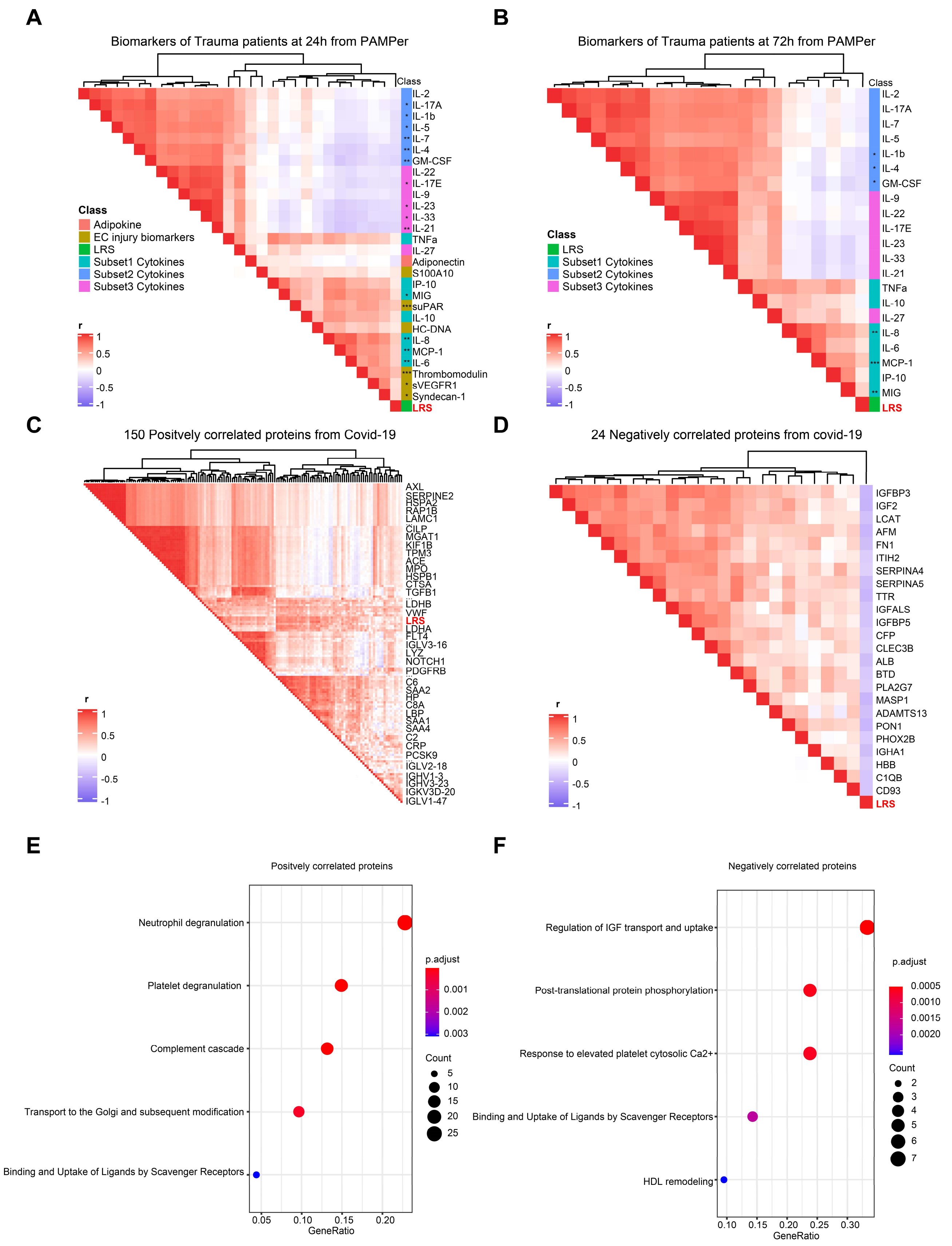

Figure S7. Association between LRS and circulating biomarkers or pathways

(A-B) Heatmap shows the correlation between LRS and circulating biomarkers at 24h and 72h after admission in trauma patients, measured by spearman correlation coefficients.

(C-D) Heatmap shows 150 positive (C) and 24 negative (D) correlating proteins with LRS in COVID-19 patients, measured by spearman correlation coefficients.

(E-F) Enriched pathways among 150 positive correlated proteins (E) and 24 negative correlated proteins (F). P value was adjusted by Benjamini-Hochberg method.

Asterisks in (A&B) indicate statistical significance for correlation coefficient. P-values are approximated by using the t distributions: *, < 0.05; **, < 0.01; ***, <0.001.
